# Characterization and Transcript Expression Analyses of Atlantic Cod *Viperin*

**DOI:** 10.3389/fimmu.2019.00311

**Published:** 2019-03-06

**Authors:** Khalil Eslamloo, Atefeh Ghorbani, Xi Xue, Sabrina M. Inkpen, Mani Larijani, Matthew L. Rise

**Affiliations:** ^1^Department of Ocean Sciences, Memorial University of Newfoundland, St. John's, NL, Canada; ^2^Division of Biomedical Sciences, Faculty of Medicine, Memorial University of Newfoundland, St. John's, NL, Canada

**Keywords:** *Gadus morhua*, *rsad2*, teleost ISGs, qPCR, dsRNA, inhibition of antiviral responses

## Abstract

Viperin is a key antiviral effector in immune responses of vertebrates including the Atlantic cod (*Gadus morhua*). Using cloning, sequencing and gene expression analyses, we characterized the Atlantic cod *viperin* at the nucleotide and hypothetical amino acid levels, and its regulating factors were investigated. Atlantic cod *viperin* cDNA is 1,342 bp long, and its predicted protein contains 347 amino acids. Using *in silico* analyses, we showed that Atlantic cod *viperin* is composed of 5 exons, as in other vertebrate orthologs. In addition, the radical SAM domain and C-terminal sequences of the predicted Viperin protein are highly conserved among various species. As expected, Atlantic cod Viperin was most closely related to other teleost orthologs. Using computational modeling, we show that the Atlantic cod Viperin forms similar overall protein architecture compared to mammalian Viperins. qPCR revealed that *viperin* is a weakly expressed transcript during embryonic development of Atlantic cod. In adults, the highest constitutive expression of *viperin* transcript was found in blood compared with 18 other tissues. Using isolated macrophages and synthetic dsRNA (pIC) stimulation, we tested various immune inhibitors to determine the possible regulating pathways of Atlantic cod *viperin*. Atlantic cod *viperin* showed a comparable pIC induction to other well-known antiviral genes (e.g., *interferon gamma* and *interferon-stimulated gene 15-1*) in response to various immune inhibitors. The pIC induction of Atlantic cod *viperin* was significantly inhibited with 2-Aminopurine, Chloroquine, SB202190, and Ruxolitinib. Therefore, endosomal-TLR-mediated pIC recognition and signal transducers (i.e., PKR and p38 MAPK) downstream of the TLR-dependent pathway may activate the gene expression response of Atlantic cod *viperin*. Also, these results suggest that antiviral responses of Atlantic cod *viperin* may be transcriptionally regulated through the interferon-activated pathway.

## Introduction

Interferon-stimulated genes (ISGs) play crucial roles as immune effectors and regulators in antiviral immune responses of fishes and other vertebrates (1, 2). As in mammals (3–5), the antiviral response of teleosts is triggered by recognizing viruses or “viral mimics” [e.g., synthetic double-stranded RNA (dsRNA): polyriboinosinic polyribocytidylic acid (pIC)] through intracellular Toll-like receptors (TLRs) or RIG-I-like receptors (RLRs), thereby activating transcription factors and enhancing the production of type I and II Interferons (IFNs) (6–9). Secreted IFNs initiate the Janus kinase-Signal transduction and activator of transcription (JAK-STAT) signaling pathway that enhances the transcription of ISGs (e.g., *viperin* and *isg15*) containing IFN gamma (IFNG)-activated sequences (GAS) and/or IFN-sensitive response elements (ISRE) in their promoters (2, 7, 10).

Virus inhibitory protein, endoplasmic reticulum (ER)-associated, IFN-inducible (Viperin), also known as Radical S-adenosyl methionine (SAM) domain-containing 2 (RSAD2) or Virus-induced gene 1 (VIG1), is an antiviral protein, highly inducible by pIC, lipopolysaccharide (LPS), viruses, and bacteria (11, 12). The expression of mammalian *viperin* is induced via IFN-dependent and independent pathways, both of which may be activated by detection of viruses or dsRNA through a member of the RLR family and activation of transcription factors (e.g., IFN regulatory factor 3, IRF3) (12–14). Mammalian Viperin localizes in the ER-derived lipid droplets and inhibits viral replication [e.g., hepatitis C virus (HCV) and influenza] (12–14). Viperin expression enhances the TLR-mediated production of type I IFN via forming a signaling complex consisting of Interleukin-1 receptor-associated kinase (IRAK1) and Tumor necrosis factor receptor-associated factor 6 (TRAF6) on lipid bodies and facilitating the nuclear translocation of IRF7 (15).

In addition to mammals, antiviral responsiveness of *viperin* was also observed in teleosts [reviewed by (16)] and an invertebrate species, i.e., Pacific oyster (*Crassostrea gigas*) (17, 18). Teleost *viperin* has been characterized and shown to be a pIC and LPS-induced gene in various species, i.e., tilapia (*Oreochromis niloticus*), annual fish (*Nothobranchius guentheri*), and red drum (*Sciaenops ocellatus*) (2, 16, 19–21). In addition, Atlantic salmon (*Salmo salar*) and crucian carp (*Carassius auratus*) *viperin* gene expression was induced in response to infectious salmon anemia virus (ISAV) (22) and grass carp reovirus (GCRV) (23), respectively, and Viperin exhibited antiviral activity against megalocytivirus in rock bream (*Oplegnathus fasciatus*) (24). As in mammals, *viperin* was shown to be an IFN-induced gene in zebrafish (*Danio rerio*) (25), and crucian carp *viperin* was suggested to be transcriptionally regulated via the RLR-activated IFN pathway (23). Although fish Viperins share some characteristics with their mammalian counterparts, the mechanisms involved in Viperin responses to immunogenic stimuli are not well-understood in fishes. Additionally, this gene/protein is not fully characterized in several teleost models.

In addition to its importance in Atlantic fisheries (26), Atlantic cod (*Gadus morhua*) exhibits a unique immune system among teleosts (27). Genomic studies have indicated that the Gadiformes lineage, including Atlantic cod, lack Major histocompatibility complex II (MHC II), CD4, Mx, and TLR5 genes, and show a unique expansion of genes including MHC I and TLR22 (28–30). Using transcriptome profiling of antiviral responses, several ISGs including *viperin* were previously identified in Atlantic cod. *viperin* displayed a strong induction in the brain of nodavirus carrier fish, the spleen and brain of pIC-injected fish, and macrophages stimulated with pIC, but not LPS (31–35). However, the full sequence, developmental and tissue expression profiles, and regulating factors of Atlantic cod *viperin* remained unknown. In this study, we aimed to fully characterize Atlantic cod *viperin*, at the nucleotide and hypothetical amino acid (AA) levels, and determine its tissue distribution, developmental expression, and signaling pathways underlying its gene expression regulation during antiviral response.

## Materials and Methods

### Gene Cloning, Sequencing, and Sequence Assembly

Gene-specific primers (GSPs) for rapid amplification of cDNA ends (RACE) were designed (see Table 1) using Primer3web v4.0.0 (http://primer3.ut.ee/) and the partial sequence of Atlantic cod *viperin* (obtained from NCBI GenBank). A pool of column-purified RNA samples from the spleens of 10 Atlantic cod injected with pIC and sampled at 24 h post-injection (HPI) (5 μg RNA per sample) was used as RNA template for the RACE cDNA synthesis [see Inkpen et al. (36) and Hori et al. (33) for experimental design].

**Table 1:** Primers used for the gene characterization and expression studies.

**Primer name**		**Primer sequence (5′-3′)**	**Application**	**Amplification efficiency (%)**	**Amplicon size (bp)**
**GENE CHARACTERIZATION PRIMERS**					
*viperin*-GSP	Forward	AGACGTCTTTCGTCCTGCCTTTGGAT	3′RACE PCR	N/A	N/A
	Reverse	CCCATGTCTGCTTTGCTCCACACATA	5′RACE PCR		
*viperin*-Nested GSP	Forward	CATCTTGGCCGTTTCCTGTGACAGTT	3′RACE PCR	N/A	N/A
	Reverse	GCATCTTCTGATTGGACTCGGGTACG	5′RACE PCR		
*viperin*-ORF GSP	Forward	AATTTGAACCATGGTGCCGG	ORF-PCR	N/A	1,060
	Reverse	TATCCATCACCACTCCAGGC	ORF-PCR		
**qPCR PRIMERS**					
*viperin*	Forward	TGTTTCCACACAGCGAAGAC	qPCR	88.7	108
	Reverse	TCCGCCAGAGAAGTTGATCT	qPCR		
*ifng*	Forward	TCGCTCTTCATGTTGGTCTG	qPCR	99.8	121
	Reverse	GGCCTTTCTGTGGATGTTGT	qPCR		
*isg15-1*	Forward	AGGACCAACAAAGGCTGATG	qPCR	88.8	110
	Reverse	CAGCCGTCCGTTAAGGTAGA	qPCR		
*lgp2*	Forward	ACAGAAGCCATCGCAGAAAT	qPCR	98.1	105
	Reverse	TTTTGCAGCACGAATCAAAC	qPCR		
*il1b*	Forward	AACACGGACGACCTGAAAAG	qPCR	93.1	126
	Reverse	GCTGATGTACCAACCGGAGT	qPCR		
*eef1a*	Forward	CAACGTCAAGAACGTCTCCA	qPCR	88.7	197
	Reverse	TGAGCTCGTTGAACTTGCAG	qPCR		
*rpl4a*	Forward	GGTGCCATACAGCTGATCCA	qPCR	94.7	123
	Reverse	CCAGGCATCACACTGCAGAA	qPCR		
*tubb2*	Forward	AGCCTGGCACTATGGACTCTGT	qPCR	91.4	129
	Reverse	GCTCGGCTCCCTCTGTGTAG	qPCR		
*eif3*	Forward	AACTGTCCGTAGTCCGCAAG	qPCR	99.7	125
	Reverse	CTGCTCAGCGAGAAACAGAA	qPCR		
*rplp1*	Forward	TCTGAAGCTAAGGCCCTCAA	qPCR	92.7	141
	Reverse	ATCGTCGTGGAGGATCAGAG	qPCR		

*GSPs, gene-specific primers; ORF, open reading frame; viperin, virus inhibitory protein, ER-associated, IFN-inducible; ifng, interferon gamma; isg15-1, interferon stimulated gene 15-1; lgp2, RNA helicase lgp2; il1b, interleukin 1, beta; eef1a, eukaryotic elongation factor 1 α; rpl4a, 60S ribosomal protein l4-a; tubb2, beta-2 tubulin; eif3, eukaryotic translation initiation factor 3; rplp1, 60S acidic ribosomal protein P1; N/A, not applicable*.

All PCR reactions in the present study were conducted on a Bio-Rad Tetrad 2 Thermal Cycler (Bio-Rad, Hercules, CA). Full-length 5′ and 3′ RACE cDNAs were synthesized using 1 μg total RNA and the SMARTer RACE cDNA Amplification Kit according to the manufacturer's instructions (Clontech, Mountain View, CA). The resulting 5′ and 3′ RACE cDNAs (i.e., 10 μl reaction) were diluted by adding 100 μl of Tricine-EDTA buffer and used for RACE PCR. A touch-down PCR [cycling program: 1 min at 95°C; 5 cycles of (94°C for 30 s, 72°C for 3 min); 5 cycles of (94°C for 30 s, 70°C for 30 s, 72°C for 3 min); 25 cycles of (94°C for 30 s, 68°C for 30 s, 72°C for 3 min); and 1 final extension cycle of 72°C for 10 min] was conducted in 50 μl reactions using the Advantage 2 Polymerase, Advantage 2 PCR buffer, dNTP Mix (Clontech), and GSPs (i.e., *viperin*-GSP; Table 1) as well as Universal Primer Mix provided by the kit (Clontech), following the manufacturer's instructions. Thereafter, 5 μl of the amplified 5′ and 3′ RACE products were diluted 50 times using Tricine-EDTA buffer and used for nested PCR. The nested PCR was performed in 50 μl reactions, using Nested GSPs (i.e., *viperin*-Nested GSP; Table 1) and Nested Universal Primer provided by the kit (Clontech), following the manufacturer's instructions. The cycling parameters for nested RACE PCR consisted of 1 min at 95°C, followed by 20 cycles of (94°C for 30 s, 68°C for 30 s, 72°C for 3 min), and 1 final extension cycle at 72°C for 10 min.

PCR products were examined on 1.2% agarose gel, and then were extracted using the QIAquick Gel Extraction kit (Qiagen) according to the manufacturer's recommendations. TA cloning of gel-extracted PCR products was performed using pGEM-T-Easy vector (Promega, Madison, WI) at 4°C overnight, using the manufacturer's instructions. Recombinant plasmids were transformed into Subcloning Efficiency DH5α Competent Cells (i.e., chemically-competent cells) (Invitrogen, Burlington, Ontario) following the manufacturer's instructions. The transformed cells were incubated in 300 μl of SOC medium (Invitrogen) for 1 h at 37°C with shaking (~225 rpm), and then cultured on Luria broth (LB)/agar plates containing 100 μg m1^−1^ ampicillin and 40 μl plate^−1^ of 40 mg ml^−1^ X-gal (Sigma, St. Louis, MO) for 16 h at 37°C. Thereafter, colonies were taken using blue/white selection and cultured in LB supplemented with ampicillin (100 μg ml^−1^) at 37°C overnight. Plasmid DNA was extracted using QIAprep Spin Miniprep Kit (Qiagen), following the manufacturer's instructions. The insert sizes of recombinant plasmids were checked using *Eco*RI (Invitrogen) digestion and agarose gel (1%) electrophoresis. Four colonies from each 5′ and 3′ RACE products were used for sequencing performed at the Genomics and Proteomics (GaP) facility, Core Research Equipment and Instrument Training (CREAIT) network, Memorial University of Newfoundland. Sequencing was conducted using an ABI 3730 DNA Analyzer with BigDye Terminator v3.1 Cycle Sequencing Kit (Applied Biosystems, Foster City, CA).

Lasergene 7.20 software (DNASTAR, Madison, WI) was used to acquire overlapping sequence from 5′ and 3′ RACE products and assemble the full-length *viperin* cDNA. GSPs amplifying the open reading frame (ORF) were designed (see Table 1) to verify the sequence assembly of full-length *viperin*. The ORF PCR was performed using the TopTaq polymerase kit (Qiagen, Mississauga, Ontario) and cDNA of pIC-stimulated Atlantic cod macrophages (i.e., pIC sub-group of no-inhibitor treatment in the macrophage stimulation experiment; see section Macrophage Isolation and Stimulation) in a 50 μl reaction. The PCR reaction was composed of 2 μl of cDNA (representing ~10 ng of input RNA; see section qPCR Assays for the cDNA synthesis method), 0.5 μM each of forward and reverse GSP, 5 μl of 10X TopTaq PCR buffer, 1.25 U of TopTaq DNA Polymerase, and 200 μM of each dNTP, with the following PCR program: initial denaturation of 3 min at 94°C, followed by 30 cycles of (94°C for 30 s, 60°C for 30 s, 72°C for 2 min), and 1 final extension cycle at 72°C for 10 min. The size of the resulting PCR product was determined by gel electrophoresis (1.2% agarose) alongside a 1 kb Plus Ladder (Invitrogen).

### Sequence Characterization and *in silico* Analyses

The AA sequence of Atlantic cod Viperin was predicted based upon assembled cDNA sequence using the SeqBuilder software of the Lasergene package (DNASTAR). To map the gene structure and determine intronic regions and genomic location, the cDNA sequence of *viperin* was aligned with genomic DNA sequence of Atlantic cod obtained from the Ensembl (http://www.ensembl.org) and Centre for Ecological and Evolutionary Synthesis (CEES: http://cees-genomes.hpc.uio.no) Genome Browsers. The AA sequences of Viperin of other fish species, as well as other vertebrate and invertebrate species (see Supplemental Table S1), were collected from the NCBI GenBank non-redundant (nr) AA database, and used for multiple sequence alignment (MSA) and phylogenetic tree construction. MSA analysis of predicted AA sequences of Atlantic cod Viperin with orthologous sequences in other species was implemented in MEGA6 software using the MUSCLE feature (37, 38). The Radical SAM domain of Atlantic cod Viperin was predicted using the PFAM database (http://pfam.xfam.org/) (39). The deduced AA sequences of Viperin homologs were aligned and used to generate a phylogenetic tree using the Neighbor-joining method in MEGA6 software (bootstrapped 10,000 times).

The neighboring genes of Atlantic cod *viperin* and its conserved synteny with other species were mapped using the Genomicus database (http://www.genomicus.biologie.ens.fr), powered by the Ensembl database. The neighboring genes and genomic location of Atlantic cod *viperin* were also confirmed using CEES Genome Browser (http://cees-genomes.hpc.uio.no).

To gain insight into transcriptional regulation of Atlantic cod *viperin*, putative transcription factor binding sites (TFBSs) were predicted in the proximal promoter region of this gene. The 1,000 bp 5′-upstream of the transcription start site of Atlantic cod *viperin* were taken from genomic DNA sequence, available at CEES Genome Browser (http://cees-genomes.hpc.uio.no/). Putative TFBSs were identified using the vertebrates' profile of the TRANSFAC database (http://genexplain.com/transfac/). This prediction was performed using the default parameters (i.e., Minimize False Positives) suggested by the TRANSFAC database, and the predicted TFBSs (core score > 0.8) with putative functions in immune responses were selected and presented herein.

### Prediction of Viperin Protein Structure

The recently-described partial mouse (*Mus musculus*) Viperin crystal structures were used as templates for homology modeling (40–43). The templates were retrieved from the Research Collaboratory for Structural Bioinformatics—Protein Data Bank (RCSB-PDB) (https://www.rcsb.org/; PDB ID: 5VSL and 5VSM). PyMOL v1.7.6 (http://www.pymol.org/) was used to visualize templates and models. Atlantic cod Viperin was computationally modeled using default parameters of I-TASSER (http://zhanglab.ccmb.med.umich.edu/I-TASSER/) (41–43). Twenty models were generated of which four models with the highest C-score (>–1.8) were further analyzed. C-score (range −5 to 2), which indicates the confidence of the quality of the predicted model, was estimated by I-TASSER. The majority of the Atlantic cod Viperin was homology-modeled based on the mouse partial Viperin crystal structure with the exception of the first 56 N-terminal AAs and the last 23 C-terminal AAs which were missing from crystal structure and, therefore, were modeled *ab initio* by I-TASSER. The same approach was used to model the full-length mouse Viperin as well as zebrafish, Atlantic salmon, and human (*Homo sapiens*) Viperins. Both [4Fe-4S] cluster and Radical SAM analog (S-adenosylhomocysteine; SAH) were manually placed in the catalytic pocket of the Atlantic cod Viperin. The surface charge and the isoelectric point of Atlantic cod and full-length mouse Viperin were estimated using the PDB2PQR server (http://nbcr-222.ucsd.edu/pdb2pqr_2.0.0/). Prediction of natively disordered regions of Viperin was conducted using the default parameters of the Protein disorder prediction server (PrDOS) website (http://prdos.hgc.jp/cgi-bin/top.cgi).

### Animals

The Atlantic cod used in this experiment were kept in two 21 m^3^ flow-through tanks (one tank for broodstock fish and one tank for fish used for tissue sampling and macrophage isolation) with optimal conditions (5.2–6.4°C, 95–110% oxygen saturation and under an ambient photoperiod) in the Dr. Joe Brown Aquatic Research Building (JBARB) of the Ocean Sciences Centre (OSC). The fish [2.29 ± 0.42 kg (mean ± SE)] used for tissue sampling and macrophage isolation were fed 3 days weekly (i.e., 1% body weight per feeding time) with a commercial diet (Skretting, BC, Canada; crude protein 50%, crude fat 18% and crude fiber 1.5%). The broodstock fish were fed mackerel, herring and squid supplemented with vitamins 2 days per week before and during spawning season. Fish (i.e., for tissue sampling and macrophage isolation experiments) were fasted 24 h and euthanized with an overdose of MS222 (400 mg L^−1^; Syndel Laboratories, Vancouver, BC) prior to the sampling. All procedures applied in the current investigation were approved by the Memorial University of Newfoundland's Institutional Animal Care Committee, according to the guidelines of the Canadian Council on Animal Care.

### Tissue Sampling

To evaluate the constitutive transcript expression of *viperin* in various tissues of adult Atlantic cod, four individuals (i.e., 2 male and 2 female) were used. Following euthanasia and dissection, samples were collected from 19 different tissues (i.e., blood, eye, brain, gill, heart, head kidney, posterior kidney, spleen, liver, gonad, stomach, pyloric caecum, midgut, hindgut, dorsal skin, ventral skin, dorsal muscle, ventral muscle, and fin) of each individual; the samples were immediately flash-frozen using liquid nitrogen and kept at −80°C until RNA extraction.

### Sampling for Developmental Series

The floating fertilized eggs were automatically collected after overnight communal spawning. Then, 1.4 L of fertilized eggs (i.e., 2-cell to ~64-cell embryos) [henceforth referred to as the zero days post-fertilization (DPF), or Day 0] were distributed into three 50 L conical incubator tanks (350 ml of eggs per tank) with 25 L h^−1^ flow rate and gentle aeration. The fertilized eggs were incubated and kept in these tanks until yolk-sac absorption stage (i.e., 20 DPF; before active feeding) at 5.5–6.1°C and under an ambient photoperiod. The developmental stage of embryos was determined daily (44). The embryos were in blastula/gastrula stages from 1 to 6 DPF (34.4 degree-days). The segmentation period began at 7 DPF (40.2 degree-days), and the golden eye stage was observed at 12 DPF (68.5 degree-days). Hatching started at 15 DPF (86.2 degree-days), and 100% of the embryos were hatched by 18 DPF (103.9 degree-days). To study *viperin* expression during early developmental stages, embryos (~0.5 ml) or larvae (~0.4 ml) were sampled daily (i.e., 0–20 DPF) from each incubator tank (i.e., one pooled sample from each replicate tank per day; *n* = 3), using 500 μm Nitex. The collected (~180 embryos or larvae per sample) samples from each tank were then flash-frozen using liquid nitrogen and kept at −80°C until RNA extraction.

### Macrophage Isolation and Stimulation

Atlantic cod macrophages were isolated from the head kidneys of 5 individuals as described in Eslamloo et al. (34, 45). All reagents (e.g., culture medium) and equipment used in this experiment were identical to Eslamloo et al. (34). Briefly, following fish dissection, the macrophage-like cells isolated from each fish were seeded into 6-well plates (Corning, Corning, NY) at an equal density of 3 × 10^7^ cells (in 2 ml L-15+) per well (16 wells per fish). The cells were cultured in Leibovitz's L-15+ [i.e., L-15 (Gibco, Carlsbad, CA) culture medium supplemented with 2 mM L-glutamine, 4.2 mM NaHCO_3_, 25 mM HEPES, 1.8 mM glucose, 100 U ml^−1^ penicillin, 100 μg ml^−1^ streptomycin (Gibco) and 1% fetal bovine serum (FBS; Gibco)] overnight at 10°C. The non-adherent cells were then removed by washing the culture dishes 3 times with L-15+.

The immune inhibitors used in this study were purchased from InvivoGen (San Diego, CA), and the stock solutions were prepared using the manufacturer's instructions. The effective doses of different immune inhibitors used in this experiment were obtained from previously published *in vitro*-based studies in fish (46–49), except for Ruxolitinib (RUX) for which dose was based on an *in vitro* study on mammalian cells (50). 2-Aminopurine (2-AP), dissolved in phosphate-buffered saline (PBS): glacial acetic acid (AcOH) (200:1) was used at 5 mM as an inhibitor of double-stranded RNA-activated protein kinase (PKR) (48). Chloroquine (CHQ), dissolved in water, was used at 80 μM as an inhibitor of endosomal TLR (49). In addition, Dimethyl sulfoxide (DMSO)-dissolved Resveratrol (RESV) and SB202190 (S90) were utilized at 50 μM for inhibiting Nuclear factor kappa-B (NFKB) and p38 Mitogen-activated protein kinase (p38 MAPK) pathways, respectively (46, 47). To inhibit JAK1/JAK2, DMSO-dissolved RUX was used at 5 μM (50). The pIC (Sigma-Aldrich) was dissolved in PBS at 10 mg ml^−1^ and used as a stock solution in the present experiment. Starting 24 h after seeding, Atlantic cod macrophages isolated from each fish were exposed to 5 inhibitors (i.e., 2 wells per inhibitor for each fish). Moreover, macrophages from each individual were subjected to PBS, DMSO (0.57 μl ml^−1^ of L-15+), or AcOH (0.17 μl ml^−1^ of L-15+) as control conditions (i.e., a total of 8 experimental groups; 2 wells per condition for each fish). The cells were incubated with inhibitors for 1 h. Afterward, macrophages from each fish under different inhibitor treatments or controls (2 wells per group) were exposed to either 50 μg ml^−1^ pIC (34) or PBS (5 μl of pIC solution or PBS ml^−1^ of L-15+). The culture medium of all groups in this study contained an identical level of PBS (i.e., 38.8 μl ml^−1^ of L-15+). The macrophages from each individual were incorporated into all experimental groups (i.e., 16 conditions in total; 5 biological replicates per group). Our previous study determined the time-dependent pIC responses of Atlantic cod macrophages (34), and based on these results the 24 h post-stimulation time point was selected for assessment of gene expression responses of macrophages in the current study. The samples were collected 24 h after pIC stimulation by removing the media and adding 800 μl TRIzol (Invitrogen, Burlington, ON) into each culture well plate. TRIzol-lysed samples were kept at −80°C until RNA extraction.

### RNA Extraction and Purification

Total RNA was extracted using TRIzol (Invitrogen) following the manufacturer's instructions. RNase-free (i.e., baked at 220°C for 7 h) ceramic mortars and pestles were used to homogenize the firm tissues (i.e., eye, gill, heart, stomach, pyloric caecum, midgut, hindgut, dorsal skin, ventral skin, dorsal muscle, ventral muscle, and fin), whereas other tissue and developmental samples were TRIzol-lysed using RNase-Free Disposable Pellet Pestles (Fisherbrand). Then, the homogenates of tissue and developmental samples were passed through QIAshredder (Qiagen) spin columns and used for RNA extraction following the manufacturer's instructions. The TRIzol-lysed macrophage samples were also processed for RNA extraction according to the manufacturer's recommendations.

Prior to purification, RNA samples (≤50 μg) in all experiments were treated with 6.8 Kunitz units of DNaseI (Qiagen) with the manufacturer's buffer (1X final concentration) for 10 min at room temperature to remove residual genomic DNA. RNA purification of adult tissue and developmental series samples (see sections Tissue Sampling and Sampling for Developmental Series) was performed using the RNeasy Mini Kit (Qiagen), whereas macrophage samples were purified using the RNeasy MinElute Cleanup Kit (Qiagen) according to the manufacturer's instructions. RNA concentration and quality were assessed using NanoDrop spectrophotometry (ND-1000), and RNA integrity was assessed by agarose gel electrophoresis (1% agarose). All column-purified RNA samples subjected to gene expression analyses in this study showed acceptable purity (i.e., A260/230 and A260/280 ratios>1.8) and integrity (i.e., tight 18S and 28S ribosomal RNA bands).

### qPCR Assays

cDNA synthesis was performed using RNA of each sample (i.e., 1 μg total RNA for macrophage samples or 5 μg total RNA for adult tissue and developmental samples), random primers (250 ng; Invitrogen), 1 μl of dNTPs (10 mM each; Invitrogen) and M-MLV reverse transcriptase (200U; Invitrogen) in the manufacturer's first-strand buffer (1X final concentration) and DTT (10 mM final concentration) at 37°C for 50 min following the manufacturer's instructions.

The qPCR assays in the current study were designed and performed on the basis of the Minimum Information for Publication of qPCR Experiments (MIQE) guidelines (51). The qPCR analyses (including an inter-plate linker and no-template controls) were conducted using a ViiA7 Real-Time PCR system (Applied Biosystems, Burlington, Ontario) in the 384-well format and a qPCR program consisting of one cycle of 50°C for 2 min, one cycle of 95°C for 10 min, and 40 cycles of (95°C for 15 s and 60°C for 1 min), followed by a dissociation curve analysis (1 cycle at 60–95°C in increments of 0.05°C per second). Fluorescence data detection occurred at the end of each cycle. qPCR assays (13 μl) were comprised of 6.5 μl Power SYBR Green Master Mix (Applied Biosystems), 50 nM of each forward and reverse primer (0.52 μl of forward and 0.52 μl of reverse primers per reaction), 1.46 μl nuclease-free water (Gibco) and 4 μl cDNA (corresponding to 4 ng of input total RNA for macrophage samples or 10 ng of input total RNA for adult tissue and developmental samples).

The primer sequences in this study were obtained from our previous study (34) (see Table 1). Prior to the qPCR assays, the primer quality tests were conducted in triplicate using a 5-point, 3-fold serial dilution of the given cDNA template [starting with cDNA representing 10 ng (macrophage experiment) or 20 ng (adult tissue and developmental experiments) of input total RNA] as well as a no-template control. The cDNA template used for primer quality tests in the macrophage experiment was a pool of 3 individual pIC-stimulated samples, whereas the template used for primer quality tests in the adult tissue and developmental studies was a pool of different adult tissues and developmental samples. Each primer pair selected for qPCR assays showed an absence of amplification in the no-template controls, an amplicon with a single melting peak (i.e., no evidence of primer dimers or non-specific products in the dissociation curve), and amplification efficiency (52) ranging from 88 to 100% (Table 1).

To identify suitable normalizer genes for qPCR assays, the expression of candidate normalizers in adult tissue [i.e., *cyclophilin a* (*cypa*), *60S acidic ribosomal protein P1*(*rplp1*), *60S ribosomal protein l4-a* (*rpl4a*), *beta-2 tubulin* (*tubb2*), *heat shock cognate 70 kDa* (*hsc70*), *eukaryotic translation initiation factor 3* (*eif3*), and *eukaryotic elongation factor 1* α (*eef1a*)], embryonic and early larval stages (*cypa, eif3, eef1a, tubb2*, and *beta-actin*) and macrophage stimulation [*protein phosphatase 1, catalytic subunit, gamma isozyme* (*ppp1cc*), *cypa, rplp1, rpl4a, tubb2, eif3*, and *eef1a*] experiments was assessed in duplicate using 50% of the samples from each experiment. The expression results were subjected to geNorm analysis using qBase software as in Eslamloo et al. (34). The normalizers expressed comparably (i.e., with lowest M-value, a measure of transcript expression stability) in samples were selected for qPCR assays of adult tissue (*eef1a, rpl4a*, and *rplp1*), embryonic and early larval development (*eif3* and *tubb2*), and macrophage stimulation (*rplp1* and *eef1a*) experiments. In addition to *viperin*, the expression levels of Atlantic cod *ifng, interferon stimulated gene 15-1* (*isg15-1*), *RNA helicase lgp2* (*lgp2*; alias *dhx58*) and *interleukin 1 beta* (*il1b*) were measured in macrophage samples. Since *ifng, isg15-1* and *lgp2* play important roles in antiviral responses (2, 16), and they showed a strong pIC induction in Atlantic cod macrophages (34), we included them in qPCR assays as positive biomarkers of antiviral responses. On the other hand, since *il1b* is an antibacterial and pro-inflammatory biomarker in fish macrophages (53), this gene was added to qPCR assays in the macrophage experiment as a negative biomarker for the inhibition of targeted pathways.

The qPCR assays for samples of each experiment were performed in triplicate using 4 ng (macrophage samples) or 10 ng (adult tissue and embryonic and early larval development samples) of input total RNA per reaction. The performance of assays between qPCR plates used in a given experiment was tested using an inter-plate linker sample [C_T_ (threshold cycle) value variations < 0.5] as well as no-template controls. ViiA7 software v1.2.2 (Applied Biosystems) was applied to calculate the relative quantity (RQ) values, relative to a calibrator sample (i.e., sample with the lowest normalized expression within each experiment) for the gene of interest, using the C_T_ values (i.e., gene of interest and normalizers) and the amplification efficiency of each primer pair (see Table 1).

The qPCR results (RQ values) of tissue and macrophage experiments were statistically analyzed using the Prism package v6.0 (GraphPad Software Inc., La Jolla, CA). The Kolmogorov-Smirnov test was performed to check the normality of the data. The transcript expression data in the macrophage experiment were analyzed using a repeated measures two-way ANOVA test designed for randomized-block experiments, whereas the transcript tissue expression data were analyzed by a one-way ANOVA test. These analyses were followed by Sidak multiple comparisons *post hoc* tests to determine the significant differences (*p* ≤ 0.05) between adult tissues as well as between and within the groups in the macrophage experiment. The qPCR results from the developmental study were not subjected to statistical analyses.

## Results

### Characterization of Atlantic Cod *Viperin* Sequence

**Figure 1:**
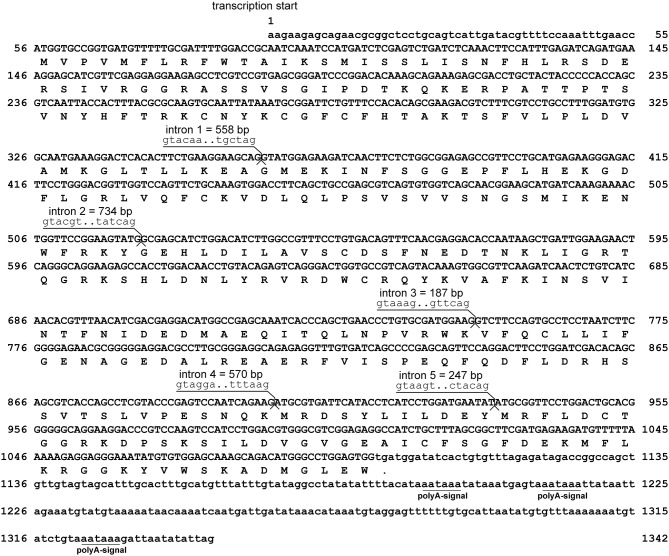
Sequence and the predicted structure of Atlantic cod *viperin*. Nucleic acid sequence (excluding poly-A tail) of *viperin* (GenBank accession: MH279971) and inferred amino acid translation. The nucleotide sequences are numbered on both sides and the inferred amino acids are shown below the coding sequence. The period shows the predicted stop codon. The lower-case letters indicate the non-coding nucleotide sequence, whereas the protein coding sequence of *viperin* is shown in upper-case letters. The intronic sequences were determined using the Ensembl (http://www.ensembl.org) and Centre for Ecological and Evolutionary Synthesis (CEES: http://cees-genomes.hpc.uio.no) Genome Browsers. Three polyadenylation signals (polyA-signal: AAUAAA) were found in the 3′UTR.

**Figure 2:**
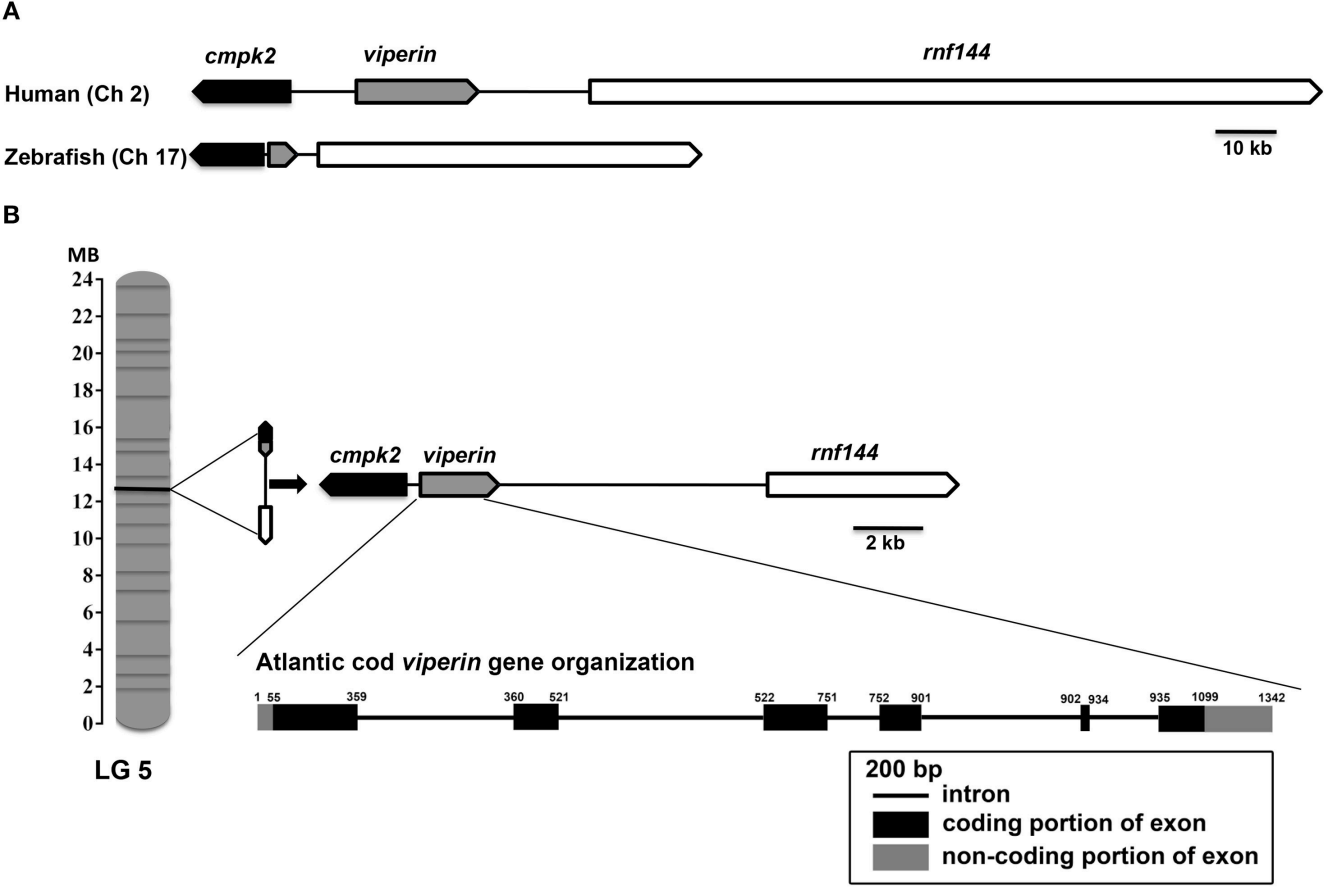
Genome organization and syntenic correspondence of Atlantic cod *viperin*. **(A)** Syntenic relationship between zebrafish and human *viperin*. **(B)** Location of *viperin* and its neighboring genes in the Atlantic cod genome, and the gene organization of Atlantic cod introns and exons. Black and gray boxes show coding (56–1099) and non-coding [5′UTR: 1-55 and 3′UTR: 1100–1342 (excluding poly-A tail)] portions of exons, respectively, and the lines connecting the exons represent introns. The synteny was determined using the Genomicus database (http://www.genomicus.biologie.ens.fr); *viperin* was found to be flanked by *cmpk2* and *rnf144* in the genomes of Atlantic cod, zebrafish and human. Arrows indicate the transcription direction of genes. Linkage Group (LG).

Assembly of RACE sequencing reads for *viperin*, validated by ORF PCR, generated a 1342 bp cDNA sequence (excluding poly-A tail) (GenBank accession: MH279971; Figure 1). As predicted using SeqBuilder, the Atlantic cod *viperin* cDNA consisted of a 55 bp 5′-UTR, 1044 bp (347 AA) ORF and a 243 bp 3′-UTR. Also, three polyadenylation signal (AAUAAA) sequences were found in the 3′-UTR (Figure 1). As determined by CEES and Ensembl databases, Atlantic cod *viperin* contained 6 exons (i.e., 1: 359 bp, 2: 162 bp, 3: 230 bp, 4: 150 bp, 5: 33 bp, and 6: 408 bp). This gene is located on Linkage Group (LG) 5 of the Atlantic cod genome (CEES, LG05:12624935-12621299). Atlantic cod *viperin* is located downstream of *cytidine monophosphate (UMP CMP) kinase 2* (*cmpk2*) and upstream of *ring finger protein 144* (*rnf144*) and shows synteny similarity to zebrafish and human. Figure 2 illustrates the genomic organization of the Atlantic cod *viperin* gene and its syntenic comparison with the *viperin* loci of zebrafish and human.

**Figure 3:**
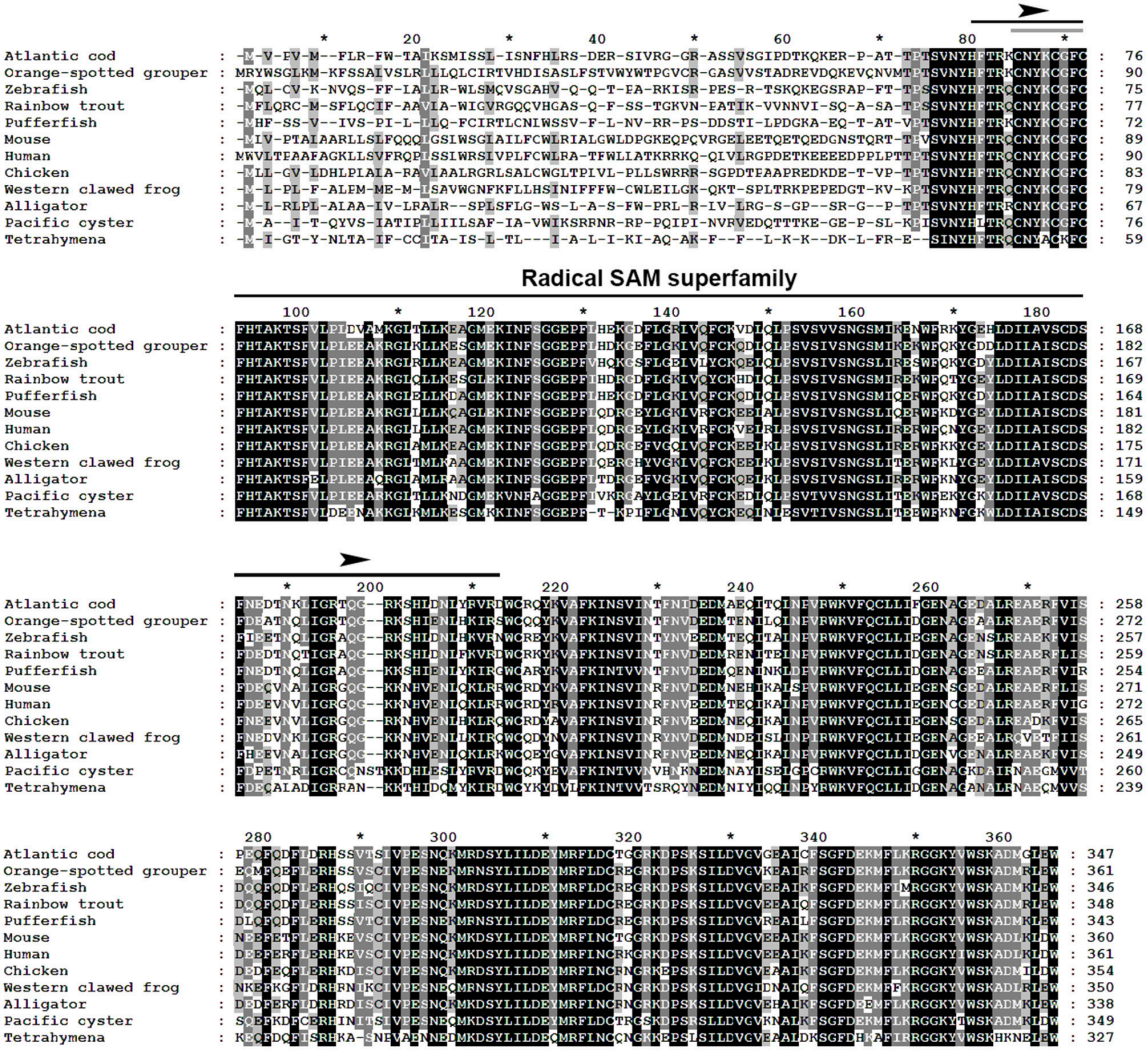
Multiple alignment of Atlantic cod Viperin deduced protein sequence with putative orthologous sequences obtained from the NCBI protein database. As identified by GeneDoc, black shading with white font shows the 100% conserved residues among the putative orthologous sequences. Dark gray shading with white font and light gray with black font denote 80 and 60% conservation among the residues, respectively. The numbers show the relative positions of the amino acid residues of Viperin putative orthologs. The Radical SAM domain of Viperin was predicted using the PFAM database (http://pfam.xfam.org/), and it includes a conserved SAM binding motif (CXXXCXXC) (i.e., marked with a gray line within the Radical SAM domain). Atlantic cod (*Gadus morhua*), Orange-spotted grouper (*Epinephelus coioides*), zebrafish (*Danio rerio*), Rainbow trout (*Oncorhynchus mykiss*), Pufferfish (*Takifugu rubripes*), Mouse (*Mus musculus*), Human (*Homo sapiens*), Chicken (*Gallus gallus*), Western clawed frog (*Xenopus tropicalis*), Alligator (*Alligator mississippiensis*), Pacific oyster (*Crassostrea gigas*), Tetrahymena (*Tetrahymena thermophila*). See Supplemental Table S1 for GenBank accession numbers and the percentage of sequence similarity to Atlantic cod Viperin.

**Figure 4:**
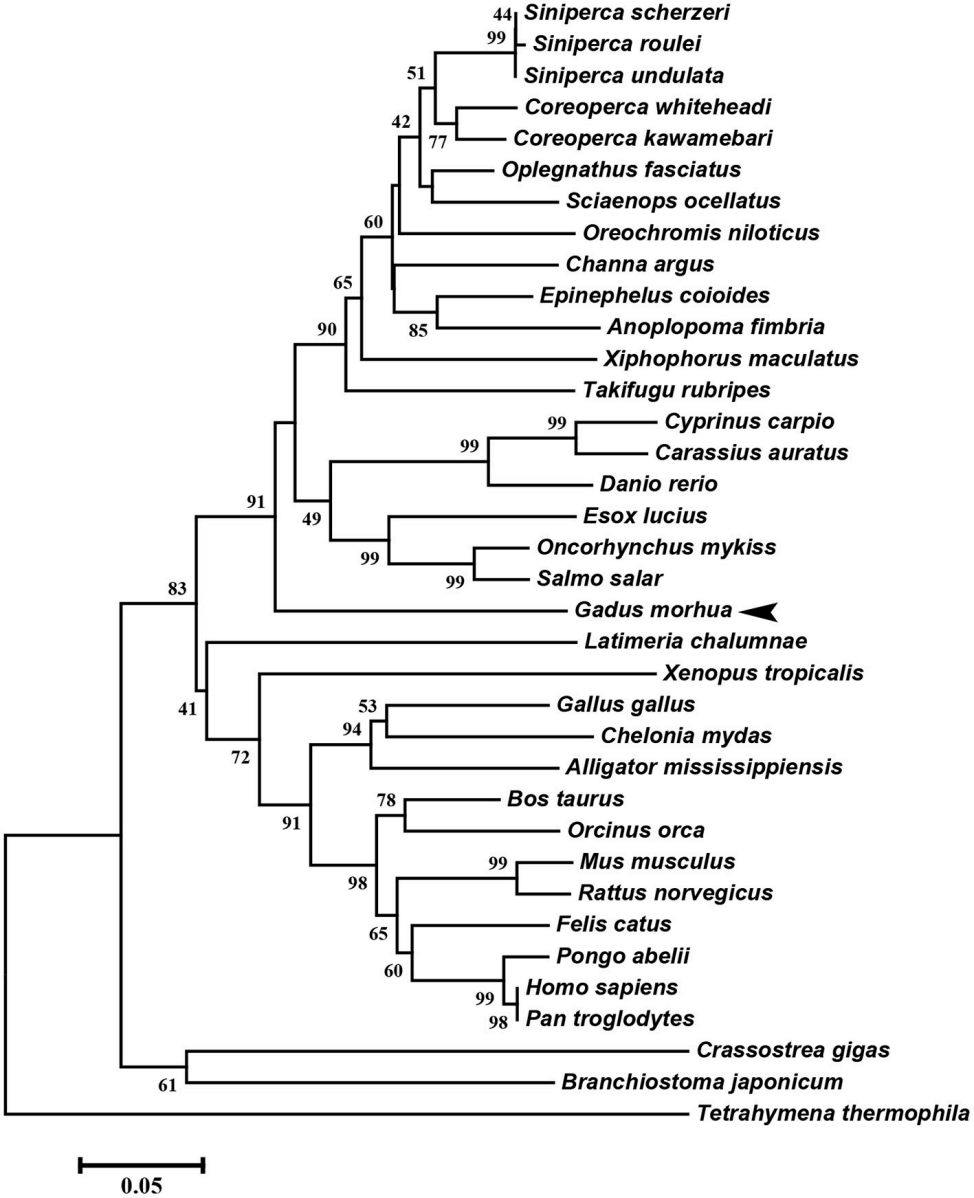
Molecular phylogenetic analysis of Viperin in various species. Putative Viperin amino acid sequence of Atlantic cod and the Viperin sequences from other species obtained from NCBI protein database were used to infer the evolutionary relationship among Viperin orthologs (see Supplemental Table S1 for GenBank accession numbers and the percentage of sequence similarity to Atlantic cod Viperin). The phylogenetic tree was generated by Neighbor-joining method and bootstrapped 10,000 times using MEGA6 software. The numbers at the branch points represent the bootstrap values. Branch lengths are proportional to calculated evolutionary distances. The scale represents number of substitutions per site. Arrowhead shows the Atlantic cod Viperin sequence.

The multiple alignment of putative AA sequences of Atlantic cod Viperin and orthologous sequences from other eukaryotic species is shown in Figure 3. This comparison revealed considerable identity (i.e., 61–82%), notably in the radical SAM domain, between Viperin of Atlantic cod and representatives of different invertebrate phyla [Ciliophora: (i.e., *Tetrahymena thermophila*) and Mollusca: (i.e., *C. gigas*)] or vertebrate classes (e.g., Actinopteri, Amphibia, Reptilia, and Mammalia). Also, the radical SAM domain of Atlantic cod Viperin contained a conserved SAM binding motif (CXXXCXXC) (see Figure 3). However, no discernible conservation in AA sequence was noted in the N-terminus of Viperin putative orthologs. The lowest percentage of similarity (i.e., 61%) was found between Viperin of Atlantic cod and the ciliated protozoan, *T. thermophila* (see Supplemental Table S1), whereas Atlantic cod Viperin showed the highest percentage of similarity to its putative orthologs in teleosts, i.e., rainbow trout (*Oncorhynchus mykiss*) (82%) and Orange-spotted grouper (*Epinephelus coioides*) (79%). The phylogenetic tree of Viperin was constructed using a MSA of putative orthologous sequences from various species (i.e., ciliated protozoan, amphioxus, mollusc, fishes, amphibian, reptiles, bird, and mammals) (Figure 4). As expected, putative orthologous sequences were grouped and sub-grouped based upon the associated phyla and classes. For example, some fish species within a given order [e.g., Salmoniformes (*O. mykiss* and *S. salar*) and Cypriniformes (*Cyprinus carpio, C. auratus*, and *D. rerio*)] were clustered together (Figure 4). Moreover, species (*O. mykiss, S. salar*, and *Esox lucius*) within the Protacanthopterygii superorder were clustered together. The Viperin of the ciliated protozoan, *T. thermophila*, formed a separate branch from the other species in the phylogenetic tree. Interestingly, Viperin of amphioxus (*Branchiostoma japonicum*) and Pacific oyster (*C. gigas*) were clustered together and separated from other species.

**Figure 5:**
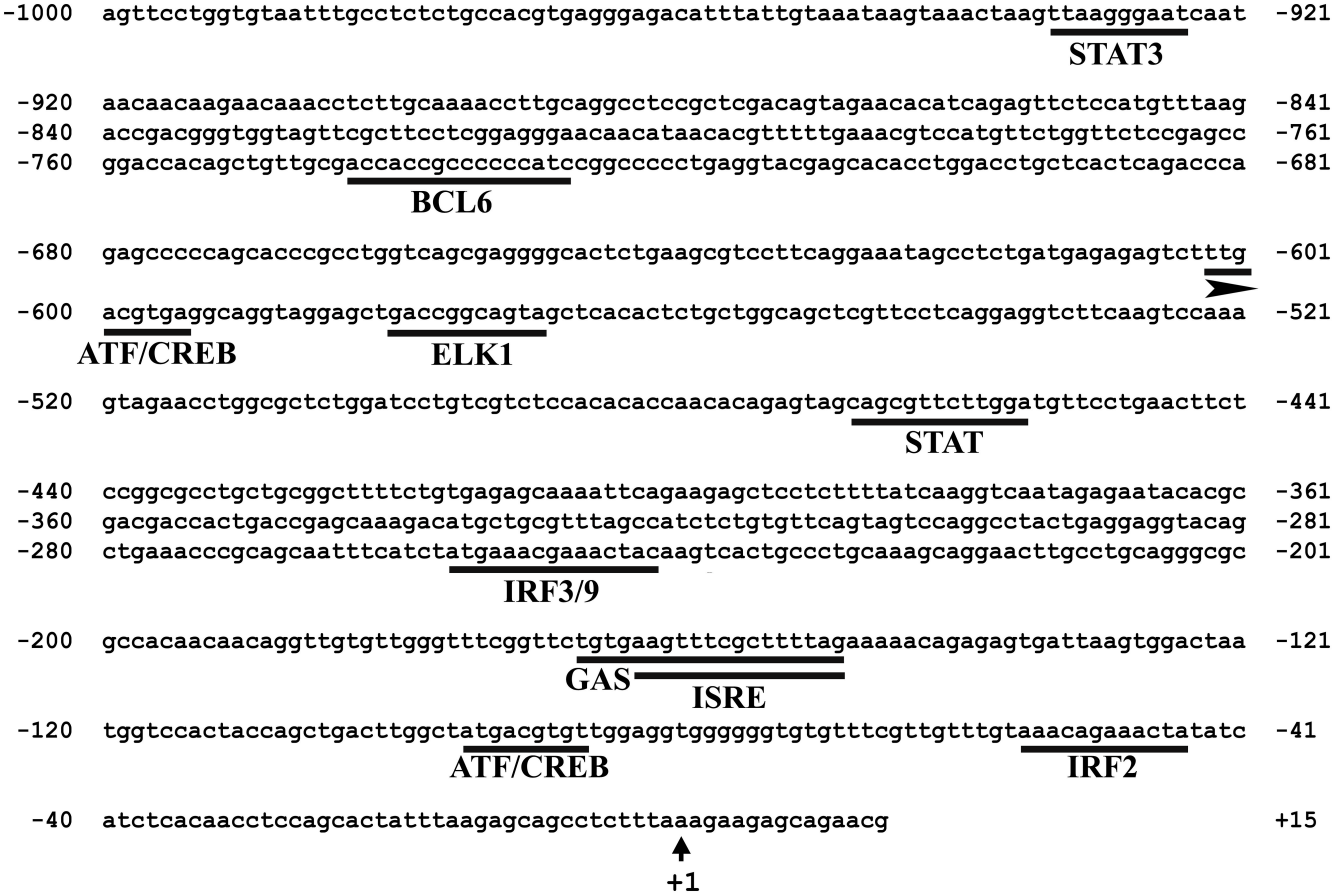
Putative immune-related transcription factor binding sites (TFBSs) predicted in the 5′-upstream region of Atlantic cod *viperin*. The proximal promoter region sequence (i.e., 1,000 bp 5′ of the transcription start site) of Atlantic cod viperin was taken from Centre for Ecological and Evolutionary Synthesis (CEES: http://cees-genomes.hpc.uio.no) Genome Browser, and TFBSs were predicted using the TRANSFAC database (http://genexplain.com/transfac/). STAT (Signal Transducer and Activator of Transcription), BCL6 (B-cell lymphoma 6), ATF (Activating Transcription Factor), CREB (cAMP Response Element-Binding protein), ELK1 (ELK1, ETS transcription factor), IRF (Interferon Regulatory Factor), GAS (IFNG-Activated Sequence), ISRE (IFN-Sensitive Response Element). +1 indicates the transcription start site of Atlantic cod *viperin*.

*In silico* analysis of the 5′-upstream region was used to predict the immune-related putative TFBSs that may play roles in regulating the expression of Atlantic cod *viperin*. As shown in Figure 5, this analysis predicted IRF3/9, STAT and Activating transcription factor (ATF) motifs located in the proximal promoter of Atlantic cod *viperin*. Moreover, GAS and ISRE, which are the binding motifs for IFNG activation factor (GAF; STAT1 homodimer) and IFN-stimulated gene factor 3 (ISGF3), respectively, were identified in the proximal promoter region of Atlantic cod *viperin*.

### Structure Prediction of Viperin Protein

**Figure 6:**
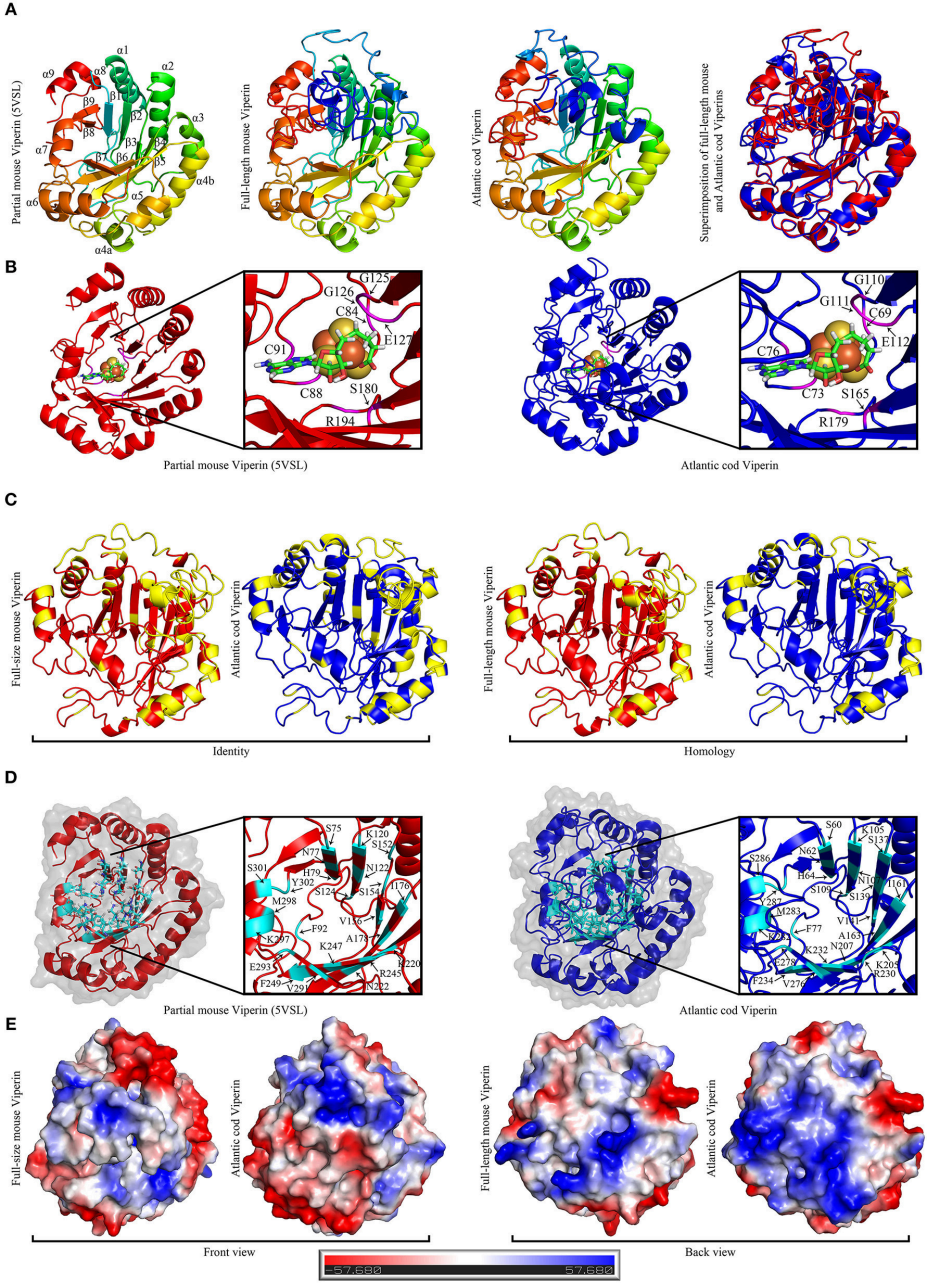
The Predicted structure of mouse and Atlantic cod Viperin. **(A)** From left to right: Representative ribbon structures of crystallized mouse Viperin (PDB ID: 5VSL); full-length mouse Viperin modeled with the additional α-helix missing from the crystal structure; predicated Atlantic cod Viperin structure; and superimposition of modeled full-length mouse (red) and Atlantic cod (blue) Viperins. In first three models, blue to red color change indicates N to C terminus progression. Loops, β-strands, and α-helices are labeled in mouse crystal structure. **(B)** Ribbon model of crystal structure of mouse Viperin (left panel) and predicted structure of Atlantic cod Viperin (right panel) showing the SAH and [4Fe-4S] cluster coordinating residues in magenta. **(C)** Comparison between the identity and homology of the mouse and Atlantic cod Viperin. Non-identical/homologous residues are shown in yellow. **(D)** Ribbon model of crystal structure of mouse Viperin (left panel) and predicted structure of Atlantic cod Viperin (right panel) showing the amino acid residues forming the catalytic cavity in cyan. **(E)** Predicted surface topology of full-length mouse and Atlantic cod Viperins. The positive, neutral, and negative residues are colored blue, white, and red, respectively.

The recently-described partial mouse Viperin crystal structures (PDB: 5VSL, 5VSM) were used as templates for homology modeling prediction of full-length Atlantic cod and mouse Viperins (Figure 6). Comparison of the predicted structure of Atlantic cod Viperin to the partial crystal structure of mouse Viperin revealed a nearly identical overall architecture, namely a partial (βα)_6_-barrel folding (Figure 6A). The CXXXCXXC or the radical SAM binding motif within which the cysteine residues ligate three iron atoms of the [4Fe-4S] cluster, and the GEE motif as well as a serine and an arginine which were shown to form hydrogen bonds with SAH, are also found to be conserved in Atlantic cod Viperin (G_125_G_126_E_127_ and S_180_ and R_194_ in mouse Viperin; G_110_G_111_E_112_ and S_165_ and R_179_ in Atlantic cod Viperin; Figure 6B) (40). Further, the aromatic residues adjacent to the third cysteine in the CXXXCXXC motif were also conserved in the Atlantic cod Viperin (F_90_ and F_92_ in mouse Viperin; F_75_ and F_77_ in Atlantic cod Viperin; Figure 3). To generate a soluble derivative of mouse Viperin for crystallization, the N-terminus region containing a suggested α-helix (residues 1 to 71) was truncated (40). Our computational modeling also suggested the conservation of this N-terminus α-helix in the Viperin of Atlantic cod, zebrafish, Atlantic salmon and human (Supplementary Figure S1). As is the case for the mouse protein (40), the Viperins of the Atlantic cod as well as the aforementioned other species were also predicted to have an intrinsically disordered N-terminal region (Supplementary Figure S2), thus allowing for a lower degree of confidence for prediction of this region.

The majority of the AA differences between the Atlantic cod and mouse Viperins are located in the α-helices and loops forming the surface of the protein, while the core of the protein forming the potential catalytic pocket cavity remained remarkably conserved (Identity = 65.1%; homology = 77.7%; Figures 6C,D). Strikingly, surface charge analysis of cod Viperin revealed that most AA differences between Atlantic cod and mouse Viperin were replacing negative/neutral AAs with more positively-charged residues, thus increasing the isoelectric point (pI) of the Atlantic cod Viperin compared to its mouse counterpart (mouse Viperin: charge at pH 7 = −5.61, pI = 5.89; Atlantic cod Viperin: charge at pH 7 = +3.78, pI = 8.61; Figure 6E).

### Constitutive Expression of Atlantic Cod *Viperin* During Early and Late Life Stages

**Figure 7:**
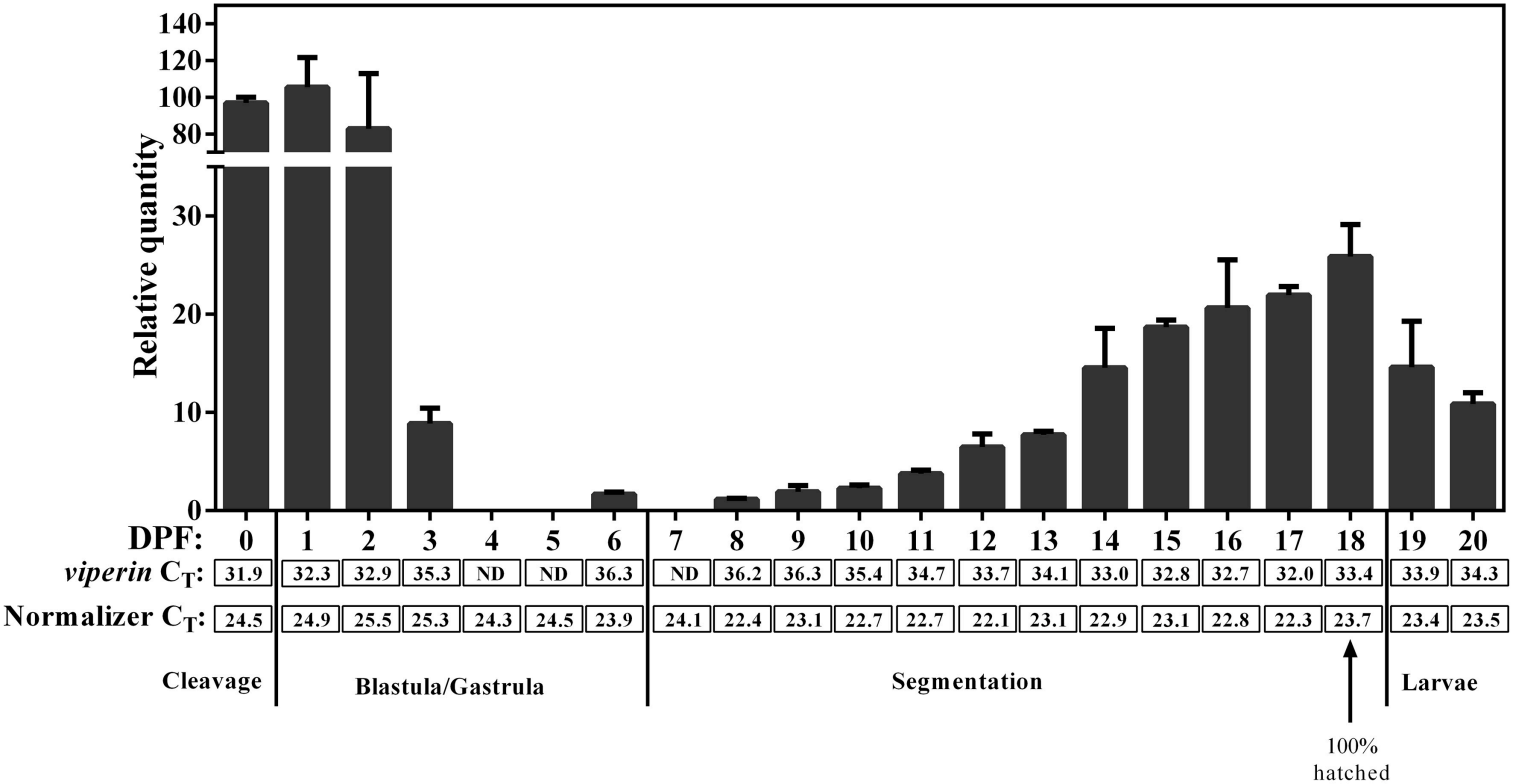
qPCR results of constitutive expression of Atlantic cod *viperin* during embryonic and early larval development. Relative quantity (RQ) data are presented as mean ± SE. The numbers below each bar show the sampling time (i.e., day post-fertilization, DPF), and 0 DPF are samples taken < 12 h after fertilization (see section Tissue Sampling). Cleavage, Blastula/Gastrula, Segmentation and Larvae describe the developmental stage at which samples were collected (44). C_T_ (Cycle threshold) values for *viperin* below each bar represent the average C_T_ of 3 pooled samples at the given time point, and ND are “not detectable” samples. Normalizer C_T_ values are the average of the geometric mean value of 2 normalizers in 3 pooled samples (see section Materials and Methods for details). These results were not statistically analyzed since the C_T_-values of the normalizers in the first 4 sampling time points were slightly higher compared with most other sampling time points.

qPCR revealed *viperin* to be a low-expressing gene (i.e., C_T_ values above 30) during early developmental stages of Atlantic cod. The expression of the normalizer genes was slightly lower (Figure 7) during very early Atlantic cod embryonic development (days 0–3), and this appeared to influence the RQ values of *viperin* for these developmental time points. Therefore, we did not subject these results to statistical analyses. However, as illustrated in Figure 7, the expression of Atlantic cod *viperin* was relatively higher from mixed cleavage until the mid-blastula stages (i.e., 0–2 DPF). Thereupon, *viperin* levels dropped to a non-detectable level during gastrula and early segmentation stages (days 4–7). In other words, Atlantic cod *viperin* was not detected at the onset of zygotic gene expression, suggesting that *viperin* is a maternal transcript. Atlantic cod *viperin* expression increased during the segmentation stage, and then appeared to decrease after hatching.

**Figure 8:**
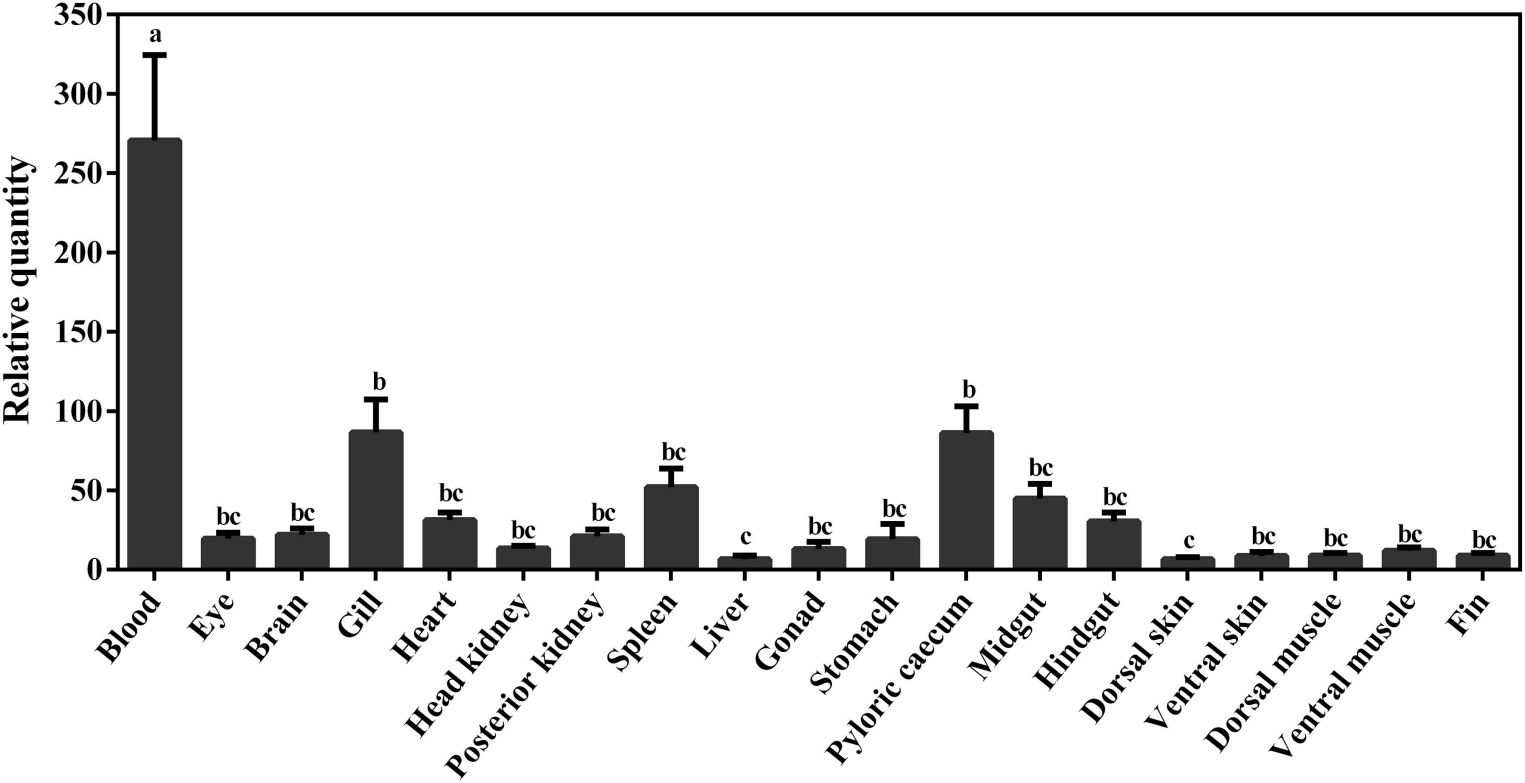
qPCR results of constitutive expression of *viperin* in different tissues of adult Atlantic cod. Relative quantity (RQ) data are presented as mean ± SE (*n* = 4). Different letters represent significant differences (*p* < 0.05) in *viperin* transcript expression between various tissues.

The constitutive expression of Atlantic cod *viperin* was assessed in 19 different adult tissues. As shown in Figure 8, the highest expression of *viperin* transcript was seen in Atlantic cod blood, which was significantly higher than all other tissues. Interestingly, the levels of *viperin* transcript in immune-related tissues, notably head kidney and spleen, were significantly lower than that in blood. Additionally, *viperin* transcript had significantly higher expression in gill and pyloric caecum compared to the dorsal skin and liver. The transcript expression of *viperin* was relatively, but not significantly, lower in the skin, muscle and fin tissues compared with some digestive tissues (e.g., midgut).

### Pathway Inhibition and *Viperin* Induction

**Figure 9:**
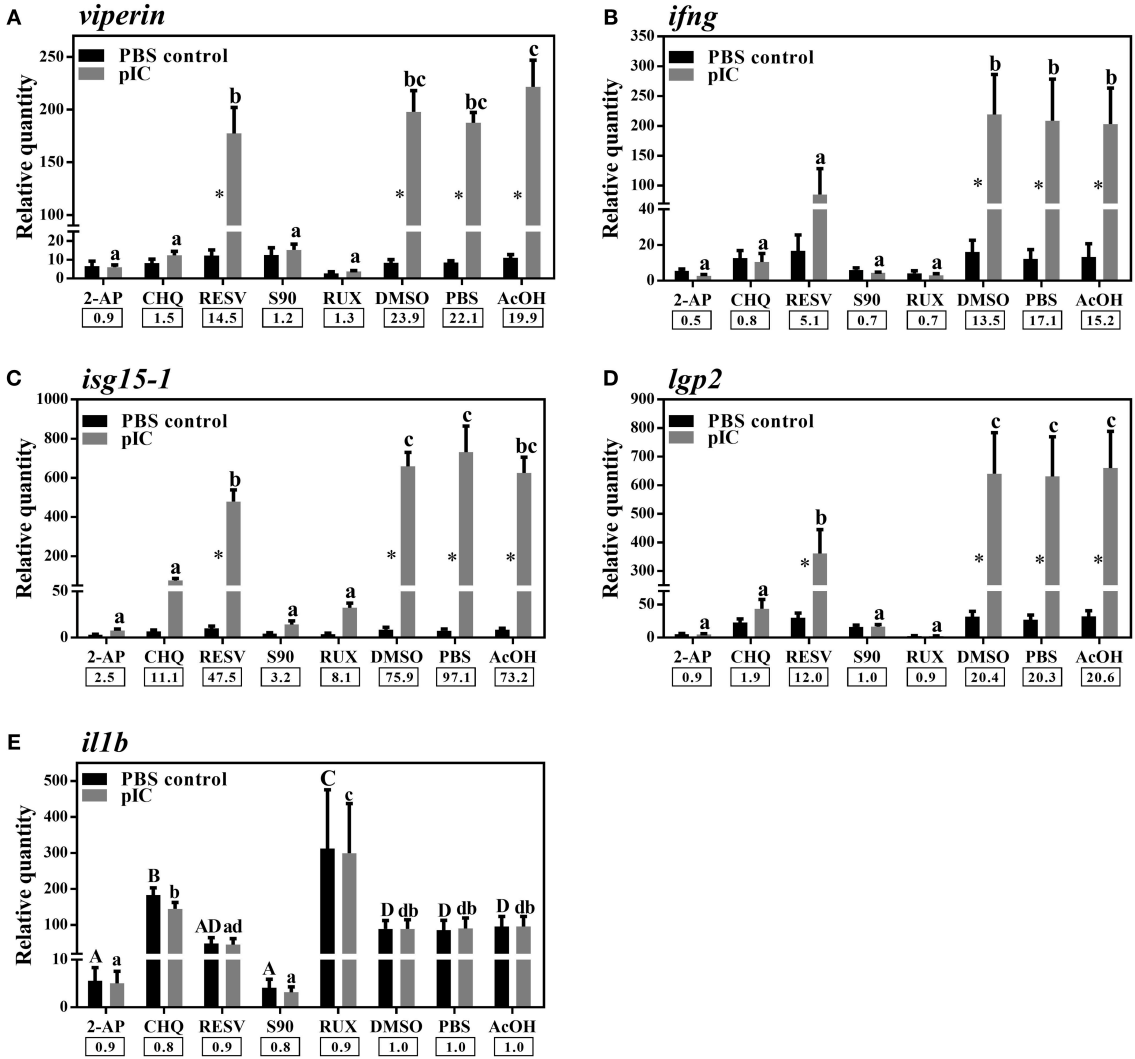
qPCR results of effects of different inhibitors on pIC induction of Atlantic cod *viperin* **(A)**, *ifng* **(B)**, *isg15-1* **(C)**, *lgp2* **(D)**, and *il1b* **(E)**. Atlantic cod macrophages were exposed to different inhibitors [i.e., 2-Aminopurine (2-AP), Chloroquine (CHQ), Resveratrol (RESV), SB202190 (S90) and Ruxolitinib (RUX) groups] or control groups [i.e., Dimethyl sulfoxide (DMSO)-, phosphate-buffered saline (PBS) and glacial acetic acid (AcOH) controls] for 1 h, and then subjected to pIC or PBS sub-groups for 24 h. DMSO group is considered as vehicle control for RESV, S90, and RUX treatments, whereas PBS and AcOH groups are considered as the control groups of 2-AP and CHQ treatments, respectively. RQ data are presented as mean ± SE. An asterisk shows significant difference (*p* < 0.05) between treatment-matched pIC and control (PBS) groups. Different letters (upper-case for PBS and lower-case for pIC) represent significant differences among PBS- or pIC-exposed inhibitor and control treatment groups. The pIC induction fold-change (pIC/control) within each treatment (i.e., inhibitors or control groups) is shown below the bars.

**Figure 10:**
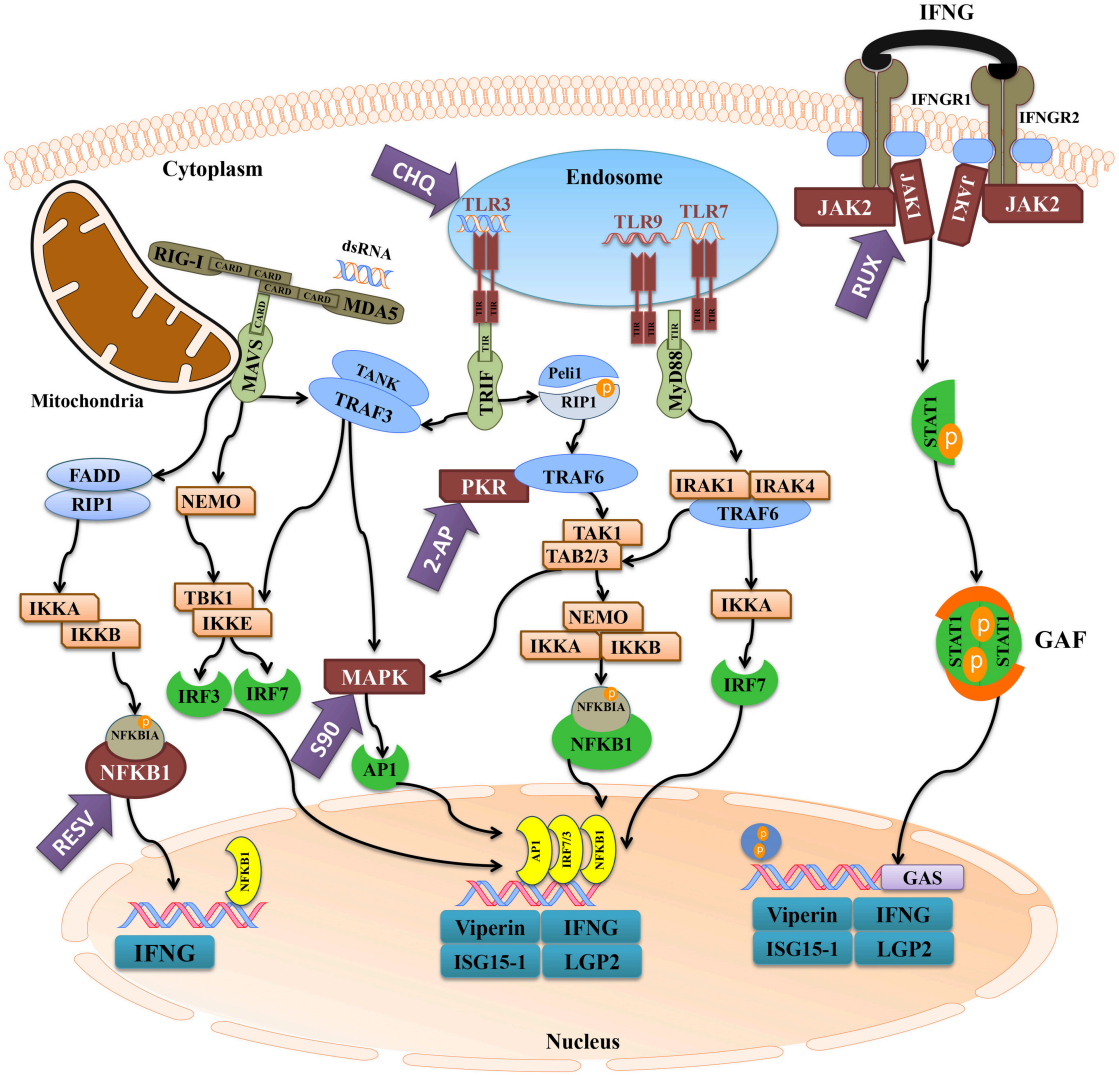
The activation of antiviral pathways in Atlantic cod. This figure was adapted from known mammalian pathways (3–5), and illustrates the effects of immune inhibitors used herein on the antiviral immune response of Atlantic cod *viperin, ifng, isg15-1*, and *lgp2*. The purple arrows represent the immune inhibitors used in this study, and the targets of inhibitors are shown in maroon. The turquoise boxes downstream of each pathway indicate the studied pIC-responsive genes that were influenced by the inhibitors. 2-AP (2-Aminopurine), CHQ (Chloroquine), RESV (Resveratrol), S90 (SB202190), RUX (Ruxolitinib), MDA5 (melanoma differentiation-associated protein 5), RIG-I (retinoic acid-inducible gene), MAVS (mitochondrial antiviral-signaling protein), FADD (FAS-associated death domain), RIP1 (receptor-interacting protein 1), IKK (NFKBIA kinase), NFKBIA (NF-kappa-B inhibitor alpha), NFKB1 (nuclear factor kappa-B 1), IFN (interferon), NEMO (NFKB1 essential modulator or IKKG), TLR (Toll-like receptor), TRIF (TIR domain-containing adaptor protein inducing IFNB), TRAF (TNF receptor-associated factor), TANK (TRAF family member-associated NFKB activator), TBK (tank-binding kinase), IRF (IFN regulatory factor), MAPK (mitogen-activated protein kinase), AP1 (transcription factor AP1), Peli1 (pellino E3 ubiquitin protein ligase 1), PKR (IFN-induced, double-stranded RNA-activated protein kinase), TAK1 [transforming growth factor beta (TGFB)-activated kinase 1], TAB (TAK1-binding protein), MyD88 (myeloid differentiation primary response gene 88), IRAK (interleukin-1 receptor-associated kinase), IFNGR (IFN-gamma receptor), JAK (Janus kinase), STAT1 (signal transducer and activator of transcription 1), GAF (IFNG-activated factor), GAS (IFNG-activated sequence), ISG15-1 (IFN-stimulated gene 15-1), LGP2 (RNA helicase LGP2).

We used different inhibitors to gain a better understanding of the signaling pathways activating the antiviral response of Atlantic cod *viperin*. As shown in Figure 9A, 2-AP, an inhibitor of the PKR-dependent pathway, significantly repressed the pIC induction of Atlantic cod *viperin* compared to the AcOH-matched control. There was no significant difference between group-matched PBS and AcOH controls, showing that *viperin* suppression in the 2-AP group was not influenced by the AcOH in which 2-AP was dissolved. CHQ also inhibited the *viperin* transcript expression in the pIC-stimulated Atlantic cod macrophages compared to the DMSO-matched vehicle control, indicating that TLR-activated pathways play an important role in the Atlantic cod *viperin* antiviral response. The expression of *viperin* transcript in pIC-treated macrophages was likewise significantly reduced in the S90 group compared to its pIC-treated DMSO vehicle control, suggesting that inhibition of the MAPK pathway strongly affects Atlantic cod *viperin* induction. Finally, our results revealed that Atlantic cod *viperin* may be a JAK1/JAK2-activated gene downstream of the IFN pathway, as there was a strong repression in the RUX-exposed pIC group compared to the pIC-treated DMSO vehicle control. On the other hand, there was no significant difference between the RESV (i.e., NFKB inhibitor) group and its DMSO-matched control. Also, with respect to the role of *ifng* in innate immune responses and *viperin* induction, the expression levels of *ifng* as well as two important IFN-induced genes, i.e., *isg15-1* and *lgp2*, were assessed in the macrophage samples (Figures 9B–D). The expression profiles of *isg15-1* and *lgp2* (Figures 9C,D) in response to various immune inhibitors were similar to *viperin*. Although there was a significant increase in the level of *isg15-1* and *lgp2* in the pIC sub-group of RESV treatment compared to its PBS control, the pIC induction of these genes in the RESV-treated samples was significantly attenuated compared to the pIC-stimulated DMSO vehicle control (Figures 9C,D). Further, Atlantic cod *ifng* showed a largely comparable expression pattern to *viperin*, and its pIC induction was significantly suppressed by 2-AP, CHQ, S90 and RUX. However, in contrast to *viperin* and similar to *isg15-1* and *lgp2*, the pIC induction of *ifng* was significantly decreased by RESV (i.e., 5.1-fold pIC induction) compared to the DMSO control group (13.5-fold pIC induction). Unlike *isg15-1* and *lgp2*, though, there was no significant difference between the PBS control and pIC sub-groups of RESV treatment for *ifng*, indicating the suppressed induction of this gene by RESV. These findings suggest that, while the expression of all genes studied herein is regulated through PKR, TLR, MAPK and IFN pathways, only *ifng* shows a significant NFKB-dependent transcriptional activation. None of the inhibitors used in this study influenced *viperin, ifng, isg15-1*, or *lgp2* expression in non-stimulated macrophages, and the inhibitors only suppressed the expression of these antiviral genes in the pIC group compared with pIC-stimulated controls (PBS, DMSO, or AcOH). This suggests that the constitutive expression of these antiviral biomarkers in Atlantic cod macrophages is regulated independent of the inhibited pathways. The expression of *il1b* was measured as an antibacterial and pro-inflammatory biomarker to examine the specificity of the immune inhibitors used in the current study. The transcription of *il1b* was not induced by pIC stimulation in any of the treatments (Figure 9E). The expression of *il1b* remained unchanged in the RESV group compared to the DMSO-matched control. Unlike the antiviral biomarkers, the constitutive expression of *il1b* was suppressed by 2-AP and S90 (Figure 9E), suggesting that PKR and MAPK pathways may have roles in regulation of *il1b* basal expression in Atlantic cod macrophages. Interestingly, *il1b* expression increased in the RUX-exposed group compared to the DMSO-matched control. Also, the expression of *il1b* was higher in PBS group of CHQ treatment compared to its PBS-matched control group. These results show that the expression profile of *il1b* is different from those of the antiviral biomarkers subjected to qPCR assays, suggesting that the inhibitors in the present study influenced their specific targets in immune pathways. In the present study, there were no significant differences between DMSO, PBS and AcOH control groups within pIC or PBS treatment, showing that the DMSO vehicle and AcOH used herein did not change the basal or pIC-induced expression of Atlantic cod *viperin, ifng, isg15-1, lgp2*, and *il1b*. Figure 10 summarizes the pathway characterization results of the current study and illustrates inhibitors, the target molecules, and their effects on the antiviral immune responses of Atlantic cod macrophages.

## Discussion

The sequencing results showed that the Atlantic cod *viperin* transcript is 1,342-bp long (excluding poly-A tail) and consists of 6 exons. Exon 5 of Atlantic cod *viperin* is the shortest, whereas exons 1 and 6 are relatively longer compared to the other exons. The transcript size of Atlantic cod *viperin* is comparable with other fish species such as red drum (21), mandarin fish (*Siniperca chuatsi*) (54), Chinese perch (*S. chuatsi*), and Ara (*Niphon spinosus*) (55). As in Atlantic cod, *viperin* transcripts of red drum (21) and human (56) were reported to include short 5′-UTRs. Similar to Atlantic cod, *viperin* genes of various vertebrates, e.g., mandarin fish (54, 55), chicken (*Gallus gallus*) (57), and mouse (*M. musculus*) (58), include 6 exons. The *viperin* exons in these species show a comparable size distribution to exons of Atlantic cod *viperin*, suggesting an evolutionarily-conserved exon/intron organization for *viperin* in vertebrates. The present study found *viperin* to be flanked by *rnf144* and *cmpk2* in the genome of Atlantic cod, which showed a conserved synteny to human and zebrafish. Previously published studies established the same gene order for *viperin* and adjacent genes of various fish (i.e., Elephant shark and tilapia), avians [i.e., chicken and zebra finch (*Taeniopygia guttata*)] and mammals [i.e., mouse and chimpanzee (*Pan troglodytes*)] (57, 59). Taken together, it seems that the genomic arrangement of *viperin* and its flanking genes, notably the opposite orientation of *viperin* and *cmpk2*, is conserved in vertebrate evolution.

As expected, the phylogenetic tree showed that the relatedness of Viperin putative orthologs appears to agree with taxonomic classification. Previous studies conducting MSAs and molecular phylogenetic analyses of Viperin in different species obtained comparable results to the current study (21, 24, 54, 59–61). Our MSA analyses revealed high levels of diversity in N-terminal AA sequences of putative orthologous Viperins among the representative species of different phyla and classes; in contrast, the radical SAM domain and the C-terminus are highly conserved.

This is the first study predicting protein structure of a teleost Viperin using crystal structure of a mammalian Viperin, and it suggests an overall structural similarity between Viperin of Atlantic cod and mouse. Specifically, the AA residues involved in coordinating the radical SAM domain as well as the [4Fe-4S] cluster of Atlantic cod were shown to be highly conserved compared to its mammalian ortholog. As in duck (*Anas platyrhynchos*) (60) and red drum (21), a conserved SAM binding motif (CXXXCXXC) was observed (see Figure 3) in the Atlantic cod Viperin. The conserved aromatic residues adjacent to the third cysteine in the CXXXCXXC motif were suggested to modulate the oxidation–reduction midpoint of the [4Fe-4S] cluster (62). The conservation of these residues in Atlantic cod Viperin seen herein suggests that the [4Fe-4S] cluster is likely used for the same function in both species. Moreover, our computational modeling revealed a catalytic cavity that is conserved between the Atlantic cod Viperin and its mammalian ortholog. Also, consistent with Viperin of human, duck, and crucian carp (60, 63, 64), our computational modeling analyses predicted the formation of an α-helix in the N-terminal region of Atlantic cod Viperin, though the N-terminus of all Viperins modeled herein are predicted to be a disordered region. Taken together, these results indicate that Atlantic cod Viperin exhibit the overall conserved structure observed/predicted in other Viperin orthologs, highly suggestive of a comparable functional role.

The radical SAM Superfamily domain is found in hundreds of proteins that play a wide range of roles (65). Viperin has been well-documented to exhibit antiviral activities against human viruses [e.g., Zika, HCV and human cytomegalovirus (HCMV)] (56, 66–68). The radical SAM domain of Viperin is a pivotal factor in the antiviral roles of this protein (67, 69). The antiviral properties of mammalian Viperin chiefly rely on the interaction of its C-terminus with viruses [HCV and Dengue Virus Type-2 (DENV-2)] (70, 71). Similar to the mammalian Viperin, the overexpression of this protein by intramuscular injection of Viperin plasmid enhanced the resistance of rock bream against megalocytivirus (24). Accordingly, the conserved antiviral activity of mammalian and teleost Viperin may be attributed to conserved SAM domains (e.g., near-identical structures of SAM domains of Atlantic cod and mouse reported herein). While N-terminal residues of mammalian Viperin were not necessary for antiviral activity of this protein, they may modulate Viperin antiviral activity (67). The N-terminal amphipathic α-helix anchors Viperin into the ER membrane and is needed for protein localization in lipid droplets (63, 72); correspondingly, the mammalian Viperin N-terminus assists the inhibition of lipid droplet-dependent viral replication [reviewed by (13)]. ER localization of the teleost Viperin was previously shown in rock bream (24) and crucian carp (64). The evolution of fish Viperin involved the positive selection of N-terminal residues (73); therefore, the positively-selected N-terminus and the conserved C-terminus of Viperin may reflect the species-dependent and ancestral functions, respectively, of this protein in antiviral responses (73). The present protein structure findings suggest that the molecular ability of teleost Viperin for binding to ER-associated lipid droplets, most likely, remained conserved, despite a large diversity between N-terminal residues of Viperin orthologs. Further studies are needed to test the correlation between diversity of N-terminal amphipathic α-helix and lipid binding and antiviral functions of teleost Viperin.

Atlantic cod *viperin* was found as a weakly-expressed gene during embryonic development. However, the expression of *viperin* was higher in mixed cleavage stage until mid-blastula (i.e., day 0–2) compared with subsequent embryonic stages (e.g., gastrula, early segmentation), showing the existence of *viperin* transcript prior to the onset of zygotic gene expression. This embryonic expression profile of *viperin* alongside its presence in adult fish gonads suggests that *viperin* is a maternal transcript in Atlantic cod. Maternal molecules (e.g., transcripts and proteins) play an important role in defense responses of fish during early life stages (74, 75). Some maternal (i.e., pre-mid-blastula expression) transcripts (e.g., *irf7, ifngr1*, and *cathelicidin*) involved in innate immune responses were previously identified in Atlantic cod (76–78). There was a considerable decrease in level of Atlantic cod *viperin* transcript after mid-blastula stage (i.e., in gastrula to early segmentation stages, 4–8 dpf). The transition from maternal to zygotic gene expression occurs at mid-blastula stage (i.e., maternal-embryo transition) (76, 79, 80). Therefore, with respect to the expression and degradation patterns of maternal transcripts (79), the non-detectable levels of Atlantic cod *viperin* immediately following mid-blastula stage may be attributed to degradation of maternal transcripts. It remains unknown if Viperin has any function in oogenesis or early embryogenesis. Nonetheless, viral hemorrhagic septicemia virus (VHSV)-induced *viperin* transcript was reported in eyed eggs and hatching fry of rainbow trout (81). Additionally, levels of *viperin* transcript increased in 48-h post-fertilization (hpf) larvae of zebrafish and 24-hpf D-veliger larvae of oyster infected with herpes simplex virus 1 (HSV-1) and Ostreid herpesvirus (OsHV-1), respectively (18, 82). We observed a steady increase in expression of Atlantic cod *viperin* transcript from early segmentation until hatch, and a somewhat decreased *viperin* expression after the hatch event. Therefore, if *viperin* function is conserved in teleost fish larvae, then its increasing constitutive expression in later stages of Atlantic cod embryonic development may provide information on the ontogeny of antiviral defense in this species. Further investigations are needed to determine whether *viperin* is a virus-responsive transcript in Atlantic cod larvae. However, similar to the *viperin* results seen herein, some immune-relevant transcripts (*cxc chemokine, interleukin 8, atf3*, and *gaduscidin-1*) of Atlantic cod were reported to increase during hatching; it was suggested that this induction may be involved in preparing cod embryos at the defensome level to combat environmental pathogens that may be encountered post-hatch (76).

This study showed that the constitutive expression of Atlantic cod *viperin* varied among different tissues. Atlantic cod *viperin* was strongly expressed in blood and, interestingly, *viperin* levels in immune-related tissues (i.e., head kidney and spleen) were significantly lower than in blood. Likewise, the expression of *viperin* in the blood of red drum (21), large yellow croaker (*Larimichthys crocea*) (83) and duck (60) was higher than other tissues, including the immune-related and hematopoietic tissues (i.e., kidney of red drum and bursa of Fabricius of duck). Head kidney is the hematopoietic site in teleost species (84), and it contains a large number of differentiating cells (e.g., myeloid progenitor cells). The tissue-dependent expression of *viperin* in vertebrates may be associated with the differentiation of immune cells, as its expression is lower in hematopoietic tissues than that in the blood. The expression of Atlantic cod *viperin* transcript in an intestinal tissue (i.e., pyloric caecum) was higher than some tissues (e.g., fin, skin, and muscle). The intestinal expression of *viperin* was previously reported in different species (Rock bream, amphioxus, and duck) (24, 59, 60). As in other vertebrates (85), the teleost intestine contains various immune cells (e.g., granulocytes and macrophages) (86), and *viperin* expression in the digestive system of Atlantic cod may be attributed to the mucosal immunity of this species. Collectively, the results suggest that some aspects of cell- and tissue-dependent expression of *viperin* may be conserved among vertebrates. However, the function of Viperin in uninfected cells and tissues remains undescribed (13), and further studies are needed to determine if the constitutive expression of *viperin* is related to its immune or potential non-immune roles.

The present study examined if different pathway inhibitors may change the pIC response of Atlantic cod *viperin* and other well-known antiviral genes (i.e., *ifng, isg15-1*, and *lgp2*). In addition to these genes, the expression of *il1b* was assessed, as a pro-inflammatory and antibacterial biomarker, to check if the inhibitors used in this study have gene-specific effects. Previous studies have documented the induction of Atlantic cod *viperin* in pIC-exposed macrophages or larval cell line (ACL cells) as well as the spleen of pIC-injected fish (31, 33, 34, 87). Similarly, pIC-triggered expression of *viperin* has been confirmed by several *in vivo*- or *in vitro*-based studies in Pacific oyster (17), crucian carp (23), red drum (21), tilapia (19), large yellow croaker (83), annual fish (20), amphioxus (59), duck (60), and mice (11). Previous studies showed no induction of Atlantic cod *viperin* in macrophages or ACL cells stimulated with different LPSs (35), as well as in spleens of Atlantic cod injected with different LPSs or formalin-killed atypical *Aeromonas salmonicida* (35, 88). We also found similar results in LPS-treated Atlantic cod macrophages (unpublished data). Accordingly, the current study did not examine the effects of inhibition of immune-related pathways on the antibacterial response of Atlantic cod *viperin*. However, antibacterial induction of vertebrate *viperin* was reported in LPS-stimulated tilapia (19) and chicken (57) as well as mouse macrophages (11) and dendritic cells (89). LPS injection slightly up-regulated *viperin* expression in the spleen of orange-spotted grouper, but *viperin* induction was stronger in response to pIC or viral (i.e., grouper iridovirus, GIV) stimulation (90). It seems that, while the transcriptional regulation of the vertebrate *viperin* by the antiviral response is conserved among vertebrates, *viperin* may have species-dependent antibacterial responses.

Figure 10 depicts the inhibitors, the targeted factors in antiviral immune responses and the summary of pathway characterization results in this study. The current investigation showed that 2-AP, CHQ, S90, and RUX significantly repressed the pIC-triggered expression of *viperin* in Atlantic cod macrophages, and similar results were seen for *ifng, isg15-1*, and *lgp2* (Figure 10). In agreement with the present study, 2-AP was previously reported to block pIC induction of *viperin* in a monocyte/macrophage-like cell line (RTS11) of rainbow trout (91) and *mx* promoter of Japanese flounder (*Paralichthys olivaceus*) embryo cells (48). 2-AP is known as an inhibitor of IFN-induced PKR autophosphorylation (92); however, a study revealed that inhibitory effects of 2-AP on IFNB transcription may occur PKR-independently, through inhibiting Akt and consequently nuclear translocation of activated IRF3 (93). The inhibitory mechanisms of 2-AP in fish species are yet to be determined, and 2-AP-associated repression of *viperin* and other studied genes in Atlantic cod may be caused by PKR- or IRF3-dependent mechanisms. In contrast to antiviral biomarker genes, 2-AP suppressed the basal expression of *il1b* in Atlantic cod macrophages. In agreement with this result, 2-AP-dependent inhibition of *il1b* expression was reported in human (94). Taken together, it seems that PKR-regulated expression of *il1b* is conserved, and 2-AP suppresses PKR-derived immune responses of Atlantic cod in a gene-specific manner. To determine TLR-dependent responses of Atlantic cod *viperin*, we used CHQ, which blocks the pIC response and TLR signaling by hindering endosomal acidification, thereby impairing PAMP recognition by intracellular TLRs (e.g., TLR3) (95). CHQ can also suppress autophagy by hindering lysosomal acidification, and it can be used as a drug with diverse functions (e.g., in malaria and cancer treatments) (96). However, immunosuppressive and antiviral activities of CHQ are associated with its roles in endosomal pH modulation and blocking nucleic acid binding to TLRs (97, 98). Therefore, the CHQ-mediated immunosuppression seen herein may be attributed to its effects on antiviral responses initiated by endosomal TLRs. CHQ was previously shown to inhibit the antiviral activity of rainbow trout macrophages (99), to decrease pIC induction of *il1b* in gilthead seabream (*Sparus aurata*) macrophages (100) and to block R848 (i.e., TLR7 ligand) response [e.g., *myeloid differentiation primary response 88* (*myd88*) and *il6*] in peripheral blood leukocytes of Japanese flounder (101). The expression of *il1b* was not influenced by pIC stimulation in the current investigation, but this gene previously showed a slight up-regulation (i.e., 1.4-fold increase at 24 h post-stimulation) in response to pIC in Atlantic cod macrophages (34). Slight differences between *il1b* results of our previous and present studies may be due to biological variability of immune responses among individuals. However, CHQ enhanced the expression of *il1b* in the control group, and this gene expression profile (i.e., diverged response of antibacterial and antiviral biomarkers) reflects the specific effects of CHQ on the antiviral response of Atlantic cod macrophages. Collectively, it seems that CHQ influences intracellular TLRs of teleosts, and that activation of Atlantic cod *viperin, ifng, isg15-1*, and *lgp2* is highly dependent upon the endosomal recognition of pIC.

S90 is known to inhibit the activity of p38 MAPK (102). S90 was found to be a strong inhibitor of LPS-induced inflammation relevant genes (e.g., *il1b* and *tnfa*) in head kidney leukocytes of Atlantic salmon (47). Generally, p38 MAPK is a well-established key regulator of inflammatory responses (103). Therefore, the suppressed constitutive expression of *il1b* in the current study may be explained by p38 MAPK-mediated regulation of inflammatory cytokines, as in a previous study involving murine macrophages (104). Nonetheless, with respect to S90-related inhibition of the virus-responsive IFNs, RIG-I-dependent p38 was suggested to be a pivotal factor in antiviral responses of mammalian dendritic cells (105). In Atlantic salmon, transcriptome profiling of antiviral responses of macrophage-like cells identified several pIC-responsive MAPKs (106). While the association of MAPK activation and antiviral responses of teleosts is not yet fully understood, our findings suggest an indirect or direct role of the p38 pathway in induction of Atlantic cod *ifng* and the other putative IFN-induced genes (i.e., *viperin, isg15-1*, and *lgp2*).

RUX blocks the activation of JAK1/JAK2 following the engagement of induced (e.g., pIC and LPS) type I and II IFNs with the IFN receptors (107, 108). As in Atlantic cod *viperin*, RUX significantly suppressed the expression of *ifng, isg15-1*, and *lgp2* in the present study, suggesting the activation of these genes downstream of JAK1/JAK2-dependent pathway. There is no report on RUX-based inhibition of IFN-dependent responses of fish species, although RUX has been reported to significantly reduce the production of IFNG in mice (50). In mammalian macrophages, RUX suppressed LPS-induced expression of IFN-regulated genes (109) and the IFN-mediated response of genes containing STAT-binding sites in their promoters (107). Similar to crucian carp *viperin* (23), the proximal promoter region of Atlantic cod *viperin* contains putative binding sites for GAS and ISRE, suggesting the IFN- or STAT-dependent regulation of this gene. Although the *viperin* putative TFBSs (i.e., GAS and ISRE sites) identified herein are compatible with the JAK1/JAK2-dependent Atlantic cod *viperin* transcript expression, these *in silico* results need to be experimentally validated by future studies. ISRE-regulated activation, as well as IFN receptor (IFNR)- and IRF-dependent induction of *viperin* have been described for mammalian macrophages (11), and chicken *viperin* transcript was found to be IFN-responsive (57). Likewise, stimulation of Atlantic salmon TO cells with recombinant IFN (110) or overexpression of IFN in zebrafish embryos (111) up-regulated the expression of teleost *viperin*. Also, induction of teleost *viperin* through activating factors downstream of MDA5 and IFN pathways was reported in crucian carp (23). In the present study, we observed a comparable gene expression profile between *viperin* and *ifng* in response to different inhibitors (except for RESV). Moreover, two putative IFN-induced genes (i.e., *isg15-1* and *lgp2*) studied herein showed expression profiles that were similar to that of *viperin*, suggesting that these genes may share signaling pathways (e.g., IFN-related pathway) activating their pIC response. Conversely, *il1b* in this study showed a different expression pattern and its expression increased in the RUX group compared to the DMSO control group. Likewise, RUX-mediated JAK inhibition increased the expression of pro-inflammatory cytokines (e.g., *il6*) in mouse macrophages (112). The qPCR results of the current study showed that RUX-mediated immune inhibition variably influenced the response of genes with different putative functions (e.g., antiviral vs. antibacterial roles) and regulatory pathways. We used RQ values of all samples from all treatments (i.e., pIC and PBS samples of inhibitor and control groups) and Pearson correlation coefficient tests to examine correlations between expression of *ifng* and other assessed genes. There were significant correlations (*p* < 0.0001) between the expression of Atlantic cod *ifng* and other antiviral genes [i.e., *viperin* (R: 0.71), *isg15-1* (R: 0.70), and *lgp2* (R: 0.94)], but no correlation was seen between *ifng* and *il1b* expression (R: 0.04; *p* = 0.74). This suggests that the IFN pathway plays important roles in transcriptional regulation of Atlantic cod *viperin, isg15-1* and *lgp2*. Additionally, the repressed antiviral response of Atlantic cod *viperin, isg15-1* and *lgp2* by other immune inhibitors (e.g., 2-AP and CHQ) may also be attributed to their influence on IFNG secretion, as *ifng* expression was significantly influenced by these inhibitors. Taken together, our results, alongside previous studies, suggest that IFN-dependent regulation of *viperin* may be conserved among vertebrates. However, a previous study suggested that the pIC induction of rainbow trout *viperin* may be independent of protein synthesis (91). Therefore, future studies using protein synthesis inhibitors and recombinant IFNs are needed to confirm IFN inducibility of Atlantic cod *viperin*.

RESV can inhibit NFKB through preventing Nuclear factor kappa-B inhibitor alpha (NFKBIA) phosphorylation (113), and it was shown to down-regulate the immune responses of head kidney leucocytes in turbot (*Scophthalmus maximus*). However, RESV did not significantly change the expression of Atlantic cod *viperin* transcript in response to pIC herein. Conversely, the pIC response of Atlantic cod *ifng* was suppressed by RESV. Also, there was a decrease in pIC induction of *isg15-1* and *lgp2* in the RESV group compared to the pIC-stimulated DMSO vehicle control group. In agreement with these results, RESV suppressed virus-induced IFNG expression in mice (114). These results suggest that the TLR- or RLR-activated NFKB may enhance the expression of Atlantic cod *ifng*, and may influence the intensity of the pIC response of *isg15-1* and *lgp2*. Although the current findings suggest NFKB-independent stimulation for Atlantic cod *viperin*, further studies using a wider range of RESV doses and multiple sampling points are needed to evaluate the time- and dose-dependent effects of RESV on Atlantic cod *viperin* expression.

In conclusion, the present study showed that Atlantic cod *viperin* is an evolutionarily conserved gene that has similar gene organization and flanking genes to its putative orthologs in other vertebrates. Atlantic cod Viperin exhibits a close phylogenetic relationship with Viperin of other teleosts. A highly-conserved protein structure reported herein suggests a functional role for the Atlantic cod Viperin comparable with that of other Viperin orthologs. Atlantic cod *viperin* transcript showed a tissue-specific constitutive expression and was most strongly expressed in the blood. The inhibitory effects of 2-AP, CHQ, and S90 on pIC induction of *viperin* transcript in Atlantic cod macrophages revealed that the expression of this gene may be dependent upon PKR, intracellular TLRs and MAPK, and/or possibly the factors (e.g., IRFs) activated downstream of these pathways. Also, RUX-associated suppression of Atlantic cod *viperin*, alongside the GAS and ISRE motifs predicted in the proximal promoter of this gene and its significant correlation with *ifng* expression in response to different immune inhibitors, suggest the IFN-mediated regulation of Atlantic cod *viperin*.

## Author Contributions

KE took a lead role in gene characterization, sequence analyses, experimental design, sampling, cell isolation, qPCR assays, data analyses, data interpretation, and the writing of manuscript draft. AG helped with RNA extraction, performed computational modeling of Viperin proteins and took part in manuscript writing. XX and SMI helped with sequence characterization, tissue sampling and RNA extraction. ML took part in data analyses and interpretation as well as manuscript writing. MLR was involved in experimental design, data analyses, and data interpretation, and took an active role in manuscript writing. All authors read and approved the final manuscript.

### Conflict of Interest Statement

The authors declare that the research was conducted in the absence of any commercial or financial relationships that could be construed as a potential conflict of interest.
