# Supplementary material for: Characterization and Transcript Expression Analyses of Atlantic Cod Viperin
Source: Front Immunol. 2019 Mar 6;10:311. doi: 10.3389/fimmu.2019.00311 (PMC6414715; doi:10.3389/fimmu.2019.00311)
Supplement: Supplementary file 1 [file Table_1.DOC]

**Supplemental Table S1:** Viperin protein of different species used in the multiple sequence alignments and phylogenetic analyses of the current study.

| **Common name** | **Description [protein name (species name)]^1^** | **GenBank accession no.** | | **Identity^2^** |
| --- | --- | --- | --- | --- |
| Japanese aucha perch | Viperin (*Coreoperca kawamebari*) | | ABO48448 | 84% |
| Barred knifejaw | Viperin (*Oplegnathus fasciatus*) | | BAM36390 | 83% |
| Japanese perch | Viperin (*Coreoperca whiteheadi*) | | ABO48449 | 83% |
| Red drum | Viperin (*Sciaenops ocellatus*) | | ADJ57695 | 82% |
| Northern snakehead | Viperin-like protein (*Channa argus*) | | AAW82780 | 82% |
| Rainbow trout | Viperin (*Oncorhynchus mykiss*) | | AAC27319 | 82% |
| Common carp | Viperin (*Cyprinus carpio*) | | AFU07734 | 82% |
| Crucian carp | IFN-inducible and antiviral protein (*Carassius auratus*) | | AAP68824 | 80% |
| Sinipercine fish | Viperin (*Siniperca undulata*) | | ABO48457 | 79% |
| Slender mandarinfish | Viperin (*Siniperca roulei*) | | ABO48446 | 79% |
| Orange-spotted grouper | Viperin (*Epinephelus coioides*) | | ACH87133 | 79% |
| Southern platyfish | PREDICTED: Radical S-adenosyl methionine domain-containing protein 2-like (*Xiphophorus maculatus*) | | XP_005801342 | 79% |
| Nile tilapia | PREDICTED: Radical S-adenosyl methionine domain-containing protein 2-like (*Oreochromis niloticus*) | | XP_003453285 | 79% |
| Leopard mandarin fish | Viperin (*Siniperca scherzeri*) | | ABO48456 | 77% |
| Coelacanth | PREDICTED: Radical S-adenosyl methionine domain-containing protein 2 (*Latimeria chalumnae*) | | XP_005998977 | 77% |
| Sumatran orangutan | Radical S-adenosyl methionine domain-containing protein 2 (*Pongo abelii*) | | NP_001129006 | 76% |
| Chicken | Viperin (*Gallus gallus*) | | ACA83729 | 75% |
| American alligator | PREDICTED: Radical S-adenosyl methionine domain-containing protein 2 (*Alligator mississippiensis*) | | XP_006275021 | 75% |
| Pufferfish | REDICTED: Radical S-adenosyl methionine domain-containing protein 2-like (*Takifugu rubripes*) | | XP_003962537 | 74% |
| Atlantic salmon | Radical S-adenosyl methionine domain-containing protein 2 (*Salmo salar*) | | ACI67411 | 74% |
| Domestic cat | PREDICTED: Radical S-adenosyl methionine domain-containing protein 2 (*Felis catus*) | | XP_003984565 | 74% |
| Green sea turtle | PREDICTED: Radical S-adenosyl methionine domain-containing protein 2 (*Chelonia mydas*) | | XP_007053248 | 74% |
| Northern pike | Radical S-adenosyl methionine domain-containing protein 2 (*Esox lucius*) | | ACO14471 | 73% |
| Sablefish | Radical S-adenosyl methionine domain-containing protein 2 (*Anoplopoma fimbria*) | | ACQ58183 | 73% |
| Zebrafish | Viperin (*Danio rerio*) | | ABJ97316 | 73% |
| House mouse | Viperin (*Mus musculus*) | | AAL50054 | 73% |
| Rat | Radical S-adenosyl methionine domain-containing protein 2 (*Rattus norvegicus*) | | NP_620236 | 73% |
| Amphioxus | Viperin (*Branchiostoma japonicum*) | | ALM30210 | 73% |
| Cattle | Radical S-adenosyl methionine domain-containing protein 2 (*Bos taurus*) | | Q2HJF9 | 71% |
| Western clawed frog | PREDICTED: Radical S-adenosyl methionine domain-containing protein 2 (*Xenopus tropicalis*) | | XP_002935073 | 71% |
| Human | Viperin (*Homo sapiens*) | | AAL50053 | 69% |
| Killer whale | PREDICTED: Radical S-adenosyl methionine domain-containing protein 2 (*Orcinus orca*) | | XP_004274956 | 69% |
| Common chimpanzee | Radical S-adenosyl methionine domain-containing protein 2 (*Pan troglodytes*) | | AFO10959 | 69% |
| Pacific oyster | Viperin (*Crassostrea gigas*) | | ALT07791 | 69% |
| Tetrahymena | Antiviral radical SAM protein viperin protein (*Tetrahymena thermophila*) | | EAS00692 | 61% |

^1^ The presented name for the associated GenBank accession number in the NCBI database.

^2^ The percentage identity of each protein sequence to Atlantic cod Viperin, as determined using Protein BLAST tool of the NCBI database.
