# Supplementary material for: Characterization and Transcript Expression Analyses of Atlantic Cod Viperin
Source: Front Immunol. 2019 Mar 6;10:311. doi: 10.3389/fimmu.2019.00311 (PMC6414715; doi:10.3389/fimmu.2019.00311)
Supplement: Supplementary file 2 [file Data_Sheet_1.PDF]

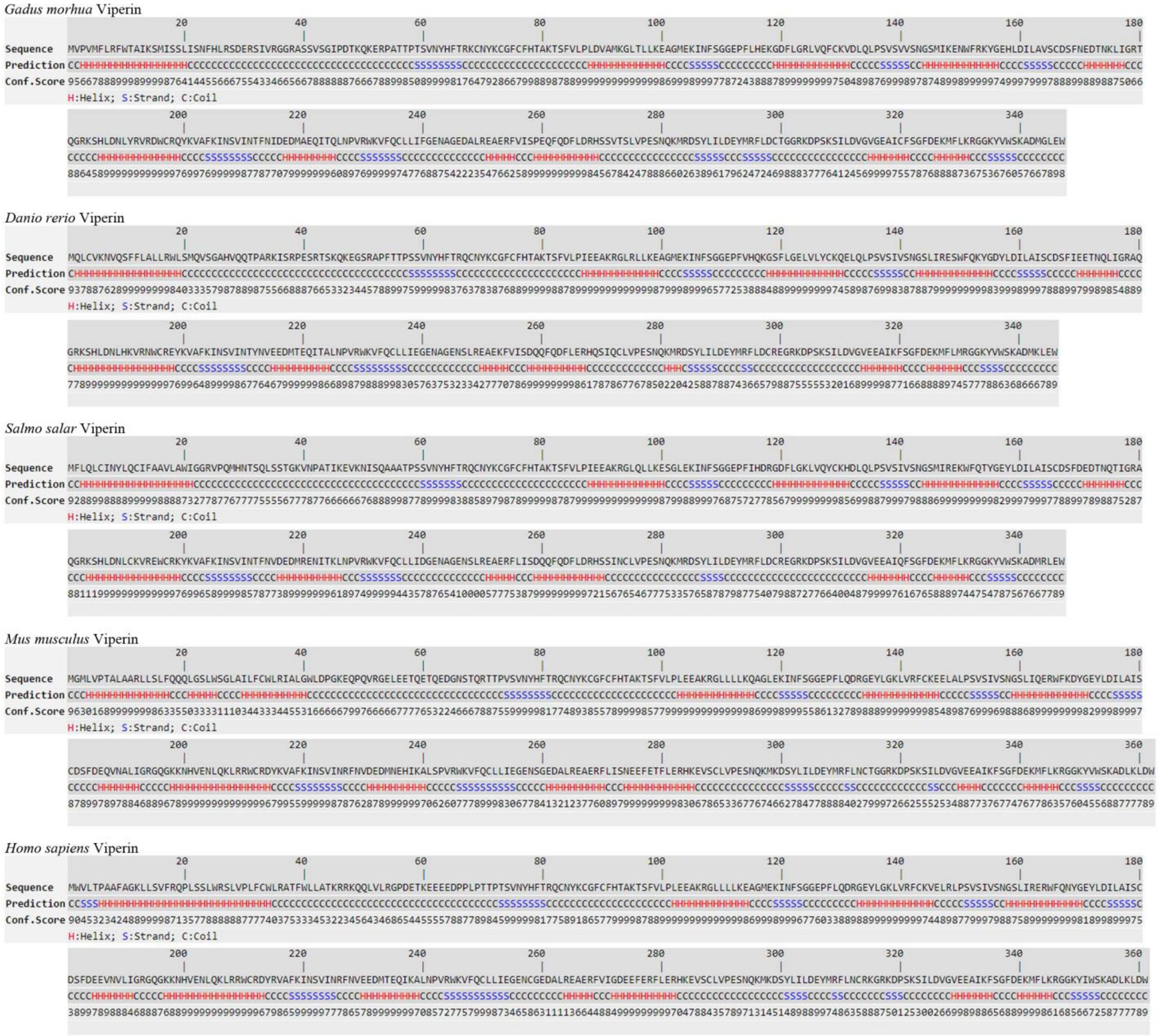

Supplementary Figure S1: Predicted secondary structure of orthologous Viperins from several species using I-TASSER website. Confidence score values range from 0 (the lowest confidence) to 9 (the highest confidence).
