# Supplementary material for: Characterization and Transcript Expression Analyses of Atlantic Cod Viperin
Source: Front Immunol. 2019 Mar 6;10:311. doi: 10.3389/fimmu.2019.00311 (PMC6414715; doi:10.3389/fimmu.2019.00311)
Supplement: Supplementary file 3 [file Data_Sheet_2.PDF]

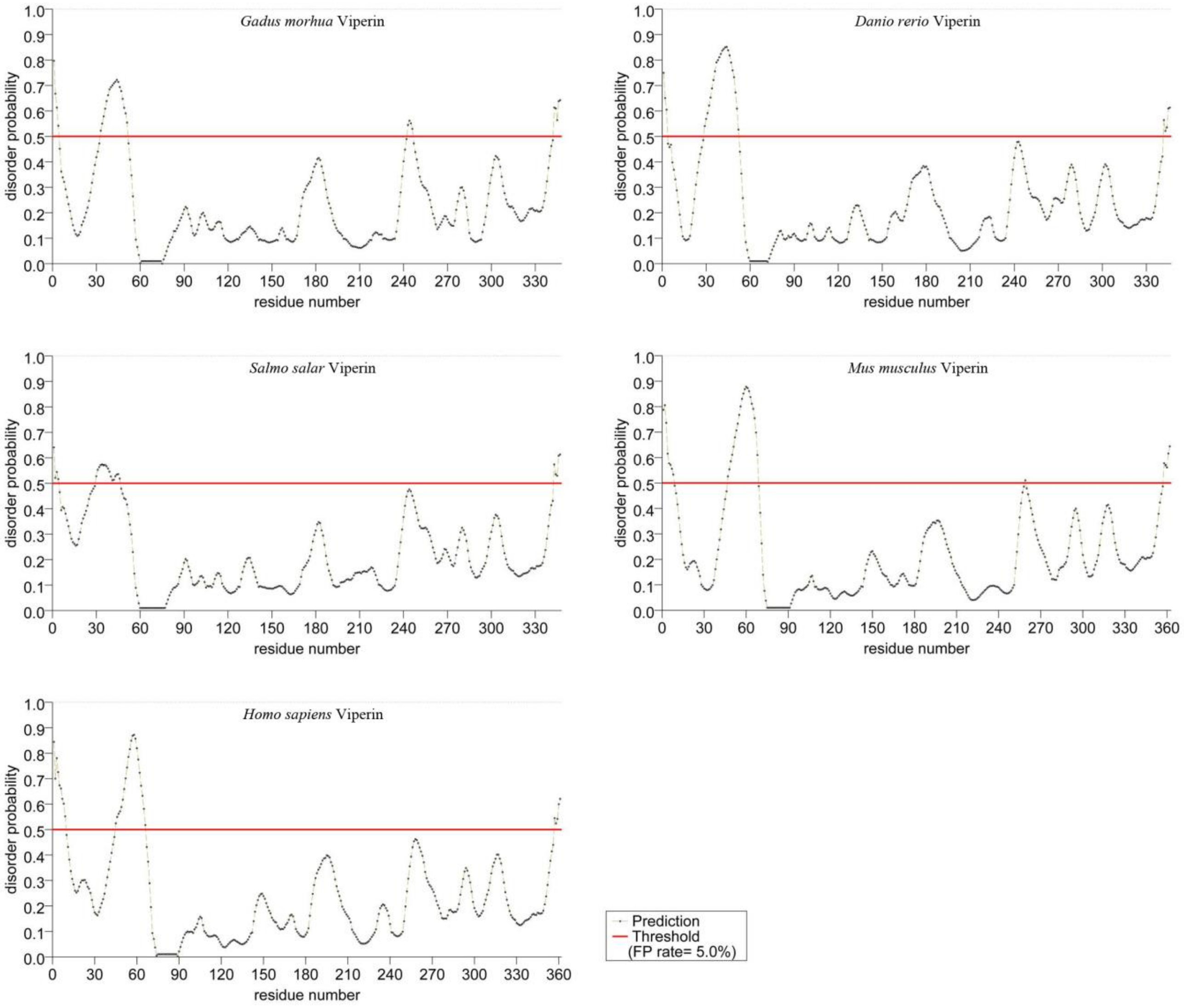

Supplementary Figure S2: Prediction of natively disordered regions of orthologous Viperins from several species. The N-terminal region of all modeled Viperins is predicted to be an intrinsically disordered region, similar to the case for the determined structure of the mouse Viperin.
